# High‐Density Oxygen Doping of Conductive Metal Sulfides for Better Polysulfide Trapping and Li_2_S‐S_8_ Redox Kinetics in High Areal Capacity Lithium–Sulfur Batteries

**DOI:** 10.1002/advs.202200840

**Published:** 2022-04-11

**Authors:** Yiyi Li, Haiwei Wu, Donghai Wu, Hairu Wei, Yanbo Guo, Houyang Chen, Zhijian Li, Lei Wang, Chuanyin Xiong, Qingjun Meng, Hanbin Liu, Candace K. Chan

**Affiliations:** ^1^ Shaanxi Provincial Key Laboratory of Papermaking Technology and Specialty Paper Development College of Bioresources Chemical and Materials Engineering Shaanxi University of Science & Technology Xi'an 710021 P. R. China; ^2^ National Demonstration Center for Experimental Light Chemistry Engineering Education Shaanxi University of Science & Technology Xi'an 710021 P. R. China; ^3^ Henan Key Laboratory of Nanocomposites and Applications Institute of Nanostructured Functional Materials Huanghe Science and Technology College Zhengzhou 450006 P. R. China; ^4^ Chongqing Institute of Green and Intelligent Technology Chinese Academy of Sciences Chongqing 400714 P. R. China; ^5^ Shaanxi Key Laboratory of Green Preparation and Functionalization for Inorganic Materials School of Materials Science and Engineering Shaanxi University of Science and Technology Xi'an 710021 P. R. China; ^6^ Materials Science and Engineering School for Engineering of Matter Transport and Energy Arizona State University Tempe 85287 USA

**Keywords:** doping, free‐standing paper, kinetics, lithium polysulfide (LiPS), lithium‐sulfur batteries

## Abstract

Exploring new materials and methods to achieve high utilization of sulfur with lean electrolyte is still a common concern in lithium‐sulfur batteries. Here, high‐density oxygen doping chemistry is introduced for making highly conducting, chemically stable sulfides with a much higher affinity to lithium polysulfides. It is found that doping large amounts of oxygen into NiCo_2_S_4_ is feasible and can make it outperform the pristine oxides and natively oxidized sulfides. Taking the advantages of high conductivity, chemical stability, the introduced large Li–O interactions, and activated Co (Ni) facets for catalyzing S*_n_* ^2–^, the NiCo_2_(O–S)_4_ is able to accelerate the Li_2_S‐S_8_ redox kinetics. Specifically, lithium‐sulfur batteries using free‐standing NiCo_2_(O–S)_4_ paper and interlayer exhibit the highest capacity of 8.68 mAh cm^–2^ at 1.0 mA cm^–2^ even with a sulfur loading of 8.75 mg cm^–2^ and lean electrolyte of 3.8 µL g^–1^. The high‐density oxygen doping chemistry can be also applied to other metal compounds, suggesting a potential way for developing more powerful catalysts towards high performance of Li–S batteries.

## Graphical abstract

Owing to its high conductivity, chemical stability, and sufficient lithium polysulfide (LiPS) affinity, the high‐density oxygen‐doped sulfide can outperform the pristine oxide and the natively oxidized sulfide in terms of catalyzing LiPS conversion for long cycling of Li–sulfur batteries.

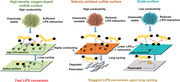

## 1 Introduction

The rechargeable lithium‐sulfur battery has attracted wide attention because of its high theoretical energy density (2600 Wh kg^−1^), low cost, and natural abundance of sulfur.^[^ ^1^ ^]^ However, achieving high energy density and long cycle life in realistic lithium‐sulfur batteries is still challenging. One of the major causes of the short cycling problem can be attributed to the well‐known “shuttle effect” of the soluble lithium polysulfide (LiPS) intermediates, which results in unexpected reactions with the lithium anode, leading to continuous sulfur loss, electrolyte consumption, and lithium anode degradation. Additionally, the low energy density issue arises because of the sluggish redox kinetics of the insulating S_8_ and its discharge products (Li_2_S_2_/Li_2_S), which result in low sulfur utilization especially in practical conditions with high areal capacity (>3 mAh cm^–2^) and lean electrolyte (electrolyte/sulfur (E/S) ratio <5 µL mg^–1^).^[^ ^2^ ^]^ So far, researchers have achieved tremendous success in tackling the “shuttle effect” and prolonging the cycling life of Li–S cells. Among these works, more and more researchers have confirmed that the “shuttle effect” can be greatly alleviated if the lithium anode is well protected by some artificial solid electrolyte interphase (SEI), interlayer, or sufficient coating.^[^ ^3^ ^]^ In this sense, if long‐term cycling of Li–S cells can be greatly improved through lithium anode protection, then more efforts should be focused on enhancing the S_8_‐Li_2_S redox kinetics to achieve high utilization of sulfur and therefore high energy density and high areal capacity Li–S batteries.^[^ ^4^ ^]^

Using highly conductive and polar transition metal compounds to adsorb LiPS and accelerate its conversion to S_8_ and Li_2_S has been considered as one of the most popular and effective ways to improve sulfur utilization in high areal capacity sulfur cathodes.^[^ ^5^ ^]^ Plenty of metal oxides,^[^ ^6^ ^]^ sulfides,^[^ ^7^ ^]^ phosphides, and nitrides have shown unique ability for catalyzing S_8_‐Li_2_S redox kinetics based on their high electronic conductivity, as well as both polar‐polar Li‐*X* (*X* = O, S, N, P) interactions and Lewis acid‐base bonding (Metal–S) with LiPS.^[^ ^8^ ^]^ Superior performance in low areal capacity Li–S cells has been achieved. However, as the areal capacity increases higher and higher, the sulfur utilization drops, and in most works, it is still challenging to push it to above 60% sulfur utilization (1000 mAh/g) with lean electrolyte (E/S < 6 µL mg^−1^) and areal capacity above 3 mAh cm^–2^.^[^ ^9^ ^]^ To achieve 3 mAh cm^–2^ and 300 Wh kg^−1^ Li–S batteries, it is critical to attain more than 60% sulfur utilization with lean electrolyte.^[^ ^10^ ^]^ With this concern, the current materials used to catalyze the S_8_–Li_2_S redox are still insufficiently effective; thus, exploring new materials and methods to achieve much higher utilization of sulfur with lean electrolyte is still needed.

The previous work has identified that most transition metal oxides are less conducting but more chemically stable and can interact with LiPS via much stronger Li–O binding compared to the metal sulfide (or nitrides, phosphide) analogs, which tend to be more conducting but chemically unstable and display weaker interactions (the Li–S(N,P) binding is less strong than Li–O) with LiPS.^[^ ^11^ ^]^ Thus, there could a possibility to combine the advantages of the oxide and sulfide (or phosphide) compounds through materials with mixed anions to enhance the catalyzing effect on S_8_‐Li_2_S redox kinetics. To fully realize the potential and benefit of the Li–S couple, it is necessary to study the doping chemistry of special compounds such as like metal oxide‐sulfides, oxide‐phosphides, oxide‐nitrides, or even phosphide‐sulfides, some of which are known but many that are yet undiscovered, which could serve as a large system of electrochemical catalysts for high‐performance Li–S batteries.^[^ ^12^ ^]^ Among these compounds containing different polyanions, oxygen‐doped (O‐doped) materials can be especially promising for improving the affinity of LiPS owing to strong Li–O binding. Recent works also show a few potential examples of O‐doped compounds (O‐doped Sb_2_S_3_,^[^ ^13^ ^]^ O‐doped VN^[^ ^14^ ^]^ and natively oxidized CoP,^[^ ^15^ ^]^ etc.) for improving the affinity of LiPS in Li–S batteries. However, yet discovered O‐doped compounds can be less conducting, and many methods that yield low‐density of O‐doping. Some compounds are natively oxidizing in the air that cannot be precisely controlled to achieve a higher oxygen content.^[^ ^15^ ^]^ It is also necessary to determine if high‐density O‐doped compounds are better at catalyzing the Li–S couples than the natively oxidized compounds. These concerns motivate us to explore a new oxygen doping method to prepare compounds with a controllable, high‐density of O‐doping, which will lead to more possibilities for developing powerful catalyst candidates for lithium‐sulfur batteries. Moreover, it is also important to obtain more understanding of the effect of O‐doping chemistry on catalyzing S_8_–Li_2_S redox reactions.

In this work, we for the first time demonstrate the preparation of high‐density O‐doped, highly conductive bimetal sulfide and study its catalyzing performance on accelerating S_8_–Li_2_S redox kinetics. We find that by controlling the degree of sulfidation, it is easy to convert spinel NiCo_2_O_4_ to cubic NiCo_2_S_4_ but also possible to make a high‐density oxygen‐doped NiCo_2_S_4_ which we call NiCo_2_(O–S)_4_. The NiCo_2_(O–S)_4_ morphology is flower‐like, similar to that of NiCo_2_O_4_ and NiCo_2_S_4_, but the crystalline structure is similar to that of cubic NiCo_2_S_4_. When NiCo_2_S_4_ is natively oxidized in air, it shows a lower density of O‐doping. However, the O and S atom ratio in NiCo_2_(O–S)_4_ can be above 1.5:1 and it can still be as electronically conducting as NiCo_2_S_4_. Taking the advantages of its high conductivity, chemical stability, strong interactions with LiPS via the polar‐polar interaction of Li–O–Co/Ni species and Co/Ni‐S bonding, the NiCo_2_(O–S)_4_ catalyst outperforms NiCo_2_O_4_ and natively oxidized NiCo_2_S_4_ in sulfur cathodes for high areal capacity (>3 mAh cm^–2^) Li–S batteries even upon long‐term cycling. Moreover, by utilizing the high‐density oxygen‐doped NiCo_2_(O–S)_4_ as an interlayer (i.e., separator coating), we achieve a high areal capacity of 8.68 mAh cm^–2^ at 1.0 mA cm^–2^ even with a sulfur loading of 8.75 mg cm^–2^ and lean electrolyte of 3.8 µL g^–1^. We also apply the high‐density O‐doping chemistry to other high conductivity and high LiPS affinity transition metal sulfides and phosphides to demonstrate the generalizability of the approach. Our work provides a potential way for preparing stable and improved electrochemical catalysts toward high sulfur utilization and high areal density sulfur cathodes.

## 2 Result and Discussion

### 2.1 Schematic and Characterization of Doping Chemistry Among NiCo_2_O_4_, NiCo_2_(O–S)_4_ and NiCo_2_S_4_

**Figure 1:**
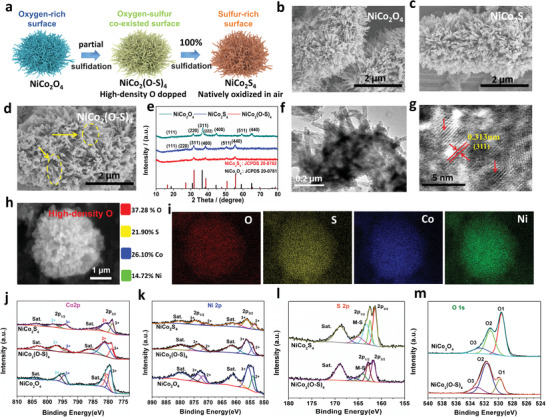
a) Schematic of sulfidation process from NiCo_2_O_4_ to NiCo_2_(O‐S)_4_ and NiCo_2_S_4_. b) Scanning electron microscopy (SEM) images of NiCo_2_O_4_ c), NiCo_2_S_4_ d) and NiCo_2_(O–S)_4_. e) X‐ray diffraction (XRD) patterns with reference patterns. f, g) Different views of transmission electron microscopy (TEM) and High‐resolution transmission electron microscopy (HRTEM) images of NiCo_2_(O‐S)_4_. h) SEM image of NiCo_2_(O–S)_4_ and corresponding EDS maps of oxygen, sulfur, cobalt, and nickel elements. XPS patterns of Co 2p (a), Ni 2p (b), S 2p (c), and O 1s (d) for NiCo_2_O_4_, NiCo_2_(O–S)_4,_ and NiCo_2_S_4_, respectively.

To study the high‐density O‐doping chemistry, NiCo_2_O_4_ was first synthesized, which can be easily converted to cubic NiCo_2_S_4_ using a simple hydrothermal sulfidation method as frequently reported before.^[^ ^16^ ^]^ By controlling the time and the quantity of the sulfidation reactant, we investigated the feasibility of preparing high‐density O‐doped NiCo_2_S_4_. Interestingly, when the amount of sulfidation reactant was insufficient (0.02 M Na_2_S, mole ratio of 1:2 for NiCo_2_O_4_: Na_2_S), the original flower‐like morphology of the NiCo_2_O_4_ appeared to have collapsed and followed be reforming of the morphology as the sulfidation time increased, which was also accompanied by a phase transformation as shown in Figure S1a–c and Figure S2a, Supporting Information. When the sulfidation time was sufficient (8 h) and the reactant quantity was increased (0.015 M, 0.02 M,0.04M, 0.2 M; mole ratio of NiCo_2_O_4_: Na_2_S ≥ 1:1.5), the NiCo_2_O_4_ also underwent a phase transformation (Figure S2b, Supporting Information). Then, when the mole concentration of Na_2_S was controlled to be 0.02 M (mole ratio of NiCo_2_O_4_: Na_2_S = 1:2, corresponding to targeted atom ratio of O:S in O‐doped NiCo_2_S_4_ can be 1:1) and 0.04 M (corresponding to targeted atom ratio of O:S in O‐doped NiCo_2_S_4_ can be 1:2), we were able to obtain high‐density O‐doped NiCo_2_S_4_ samples with flower‐like morphology assembled by 1D nanofibers, similar to that in NiCo_2_O_4_ and NiCo_2_S_4_, but with the same crystal structure as cubic NiCo_2_S_4_ as illustrated in Figure S1d,e,f, and Figure S2b, Supporting Information. **Figure** **1**a shows the schematic of the sulfidation process from NiCo_2_O_4_ to NiCo_2_S_4_. As the sulfidation amount and concentration are carefully controlled, the high‐density O‐doped NiCo_2_S_4_ samples, marked as NiCo_2_(O–S)_4_ (with 0.02 M Na_2_S, Sample 1) and S2‐NiCo_2_(O–S)_4_ (with 0.04 M Na_2_S, Sample 2), can be prepared as mentioned above and characterized as following. The scanning electron microscopy (SEM) images in Figure 1b‐d, Figure S1a,d–i, Supporting Information, and TEM image in Figure 1f also indicate that these materials share a similar morphology with a 3D flower‐like structure. It should be emphasized that similar morphologies can enable a better investigation of the effect of composition on the electrochemical performance. Figure 1e and Figure S2b, Supporting Information show the X‐ray diffraction (XRD) patterns of these materials, which indicates that the structure of the as‐synthesized NiCo_2_O_4_ and NiCo_2_S_4_ particles are in good agreement with spinel NiCo_2_O_4_ and cubic NiCo_2_S_4_, respectively, while NiCo_2_(O‐S)_4_ clearly contains the main NiCo_2_S_4_ phase but with lower intensity reflections than those in S2‐NiCo_2_(O–S)_4_ and NiCo_2_S_4_, which somehow is owing to its transition‐state towards pure NiCo_2_S_4_.^[^ ^17^ ^]^ It is also verified by Figure S3, Supporting Information, that the synthesized NiCo_2_(O‐S)_4_ and S2‐NiCo_2_(O–S)_4_ are O‐doped compounds but not simple mixtures of separated phases of NiCo_2_O_4_ and NiCo_2_S_4_. High‐resolution transmission electron microscopy (HRTEM) images (Figure 1g and Figure S4, Supporting Information) of the NiCo_2_(O–S)_4_ reveal distorted lattice fringes with spacings corresponding to (311) planes slightly larger than the theoretical spacings for NiCo_2_S_4_, while the (400), (511) and (440) spacings are slightly lower (compared in Table S1, Supporting Information). These differences in lattice spacings in NiCo_2_O_4_, NiCo_2_(O–S)_4_ and NiCo_2_S_4_ could be due to the introduction of oxygen and sulfur vacancies, which may cause defects and rearrangement of the atoms in the lattices.^[^ ^18^ ^]^ Furthermore, energy‐dispersive spectroscopy (EDS) and electron energy loss spectroscopy (EELS) were applied for verifying the high‐density O‐doped states of NiCo_2_(O–S)_4_ and S2‐NiCo_2_(O–S)_4_. Figure 1h shows that O, S, Ni, and Co are uniformly distributed with a particular atomic percentage of 14.72% Ni, 26.10% Co, 21.90% S, and 37.28% O, which is roughly the same as the theoretical ratio of 1(Ni):2(Co):4(O+S). Additional EELS mapping and spectra (Figure S5 and Figure S6, Supporting Information) also confirm the uniform and high‐density O‐doped state of the NiCo_2_(O–S)_4_ surface, indicating the O and S atom ratio can be above 1.5:1. Specifically, Figure S7, Supporting Information shows NiCo_2_S_4_ is natively oxidized in the air with a low concentration of O‐doping, which is similar to natively oxidized sulfides and phosphides reported in Wang's work.^[^ ^15^ ^]^ The atomic percentage of oxygen in natively oxidized NiCo_2_S_4_ is quite low (7.60%, O:S = 1:7). However, by simply controlling the concentration of Na_2_S and sulfidation time, the O‐doping content can be increased to 15.6% for S2‐NiCo_2_(O‐S)_4_ (O:S = 1:3) and finally to 37.28% O (O:S = 1.7:1) for NiCo_2_(O‐S)_4_ as shown in Figure S8 and Figure S9, Supporting Information. In order to investigate the air stability of these samples with different amounts of O‐doping, XRD was carried out after exposing NiCo_2_O_4_, NiCo_2_(O–S)_4_, and natively oxidized NiCo_2_S_4_ powders to ambient air for one month. Surprisingly, the XRD patterns after the air exposure (Figure S10a, Supporting Information) show that NiCo_2_S_4_ completely degraded and no phase changes were observed for NiCo_2_(O‐S)_4_ and NiCo_2_O_4_. Further exposing S2‐NiCo_2_(O‐S)_4_ and NiCo_2_(O‐S)_4_ (Figure S10b, Supporting Information) for more days of air exposure, the XRD patterns and optical images of these samples show that S2‐NiCo_2_(O‐S)_4_ starts to be partially degraded after 40 days and totally degraded after 70 days, while NiCo_2_(O‐S)_4_ shows a lower degradation rate than S2‐NiCo_2_(O‐S)_4_, suggesting that high‐density O‐doping greatly increases the stability (mainly against oxidation) of the sulfides and can be a potential strategy for other sulfides or phosphides. Noting that NiCo_2_(O‐S)_4_ shows the highest amount of O‐doping (37.28%) and is more stable in the air during extended storage compared to S2‐NiCo_2_(O‐S)_4_ (15.60%), we focused on NiCo_2_(O‐S)_4_ for subsequent characterization and comparison. Based on the greatly increased oxygen‐content in high‐density O‐doped NiCo_2_(O‐S)_4_ (37.28%) compared to natively oxidized NiCo_2_S_4_ (less than 10.0%), we can elucidate the role of oxygen content on the properties of these compounds for catalyzing Li‐S redox reactions compared to the sulfur‐free NiCo_2_O_4_.

X‐ray photoelectron spectroscopy (XPS) was also conducted to analyze the changes in chemical valence states of the NiCo_2_O_4_, NiCo_2_(O–S)_4,_ and NiCo_2_S_4_ powders. The Co 2p and Ni 2p peaks in NiCo_2_(O–S)_4_ are similar to those in NiCo_2_S_4_, confirming that they share similar chemical states (Figure 1j,k). The S 2p spectra of NiCo_2_(O–S)_4_ as shown in Figure 1l also exhibit a lower intensity than NiCo_2_S_4,_ indicating a lower sulfur content on NiCo_2_(O‐S)_4_ surface. In Figure 1m, the O1 (*M*–O bond, where *M* = metal) peak is slightly shifted to higher binding energies in NiCo_2_(O–S)_4_ compared to NiCo_2_O_4_ while the intensity is lower, which could be due to the weakened *M*–O bond after the substitution of oxygen atoms with sulfur. All the above data confirm that the partial sulfidation process of NiCo_2_O_4_ results in the co‐existence of sulfur and oxygen in the cubic NiCo_2_S_4_ structure and the high‐density O‐doped NiCo_2_S_4_ can be obtained as we call NiCo_2_(O–S)_4_. To further understand the effect of oxygen doping on the structure and property changes of pure NiCo_2_S_4_, we used density functional theory (DFT) to calculate the (311) surface structure as shown in Figure S11a,b, Supporting Information. The calculated total density of states (DOS) showed that both NiCo_2_S_4_ and one O‐atom doped NiCo_2_S_4_ (O‐NiCo_2_S_4_) exhibit metallic properties, which should be beneficial for electronic transport (Figure S11c,d, Supporting Information). Although there is no marked difference in the DOS of pristine NiCo_2_S_4_ and O‐NiCo_2_S_4_ (311), the d‐band center of O–NiCo_2_S_4_ (311) is −1.3955, −1.2858, and −1.3407 for the spin‐up, spin‐down and average value, respectively, slightly lower than that of pristine NiCo_2_S_4_(311) (−1.392, −1.278 and −1.335 for spin‐up, spin‐down and average value, respectively). The location of the d‐band center further under the Fermi level implies the potential ability for trapping small molecules on the O–NiCo_2_S_4_ (311) surface.

### 2.2 Conductivity and Chemical Interaction with LiPS of NiCo_2_O_4_, NiCo_2_(O–S)_4_ and NiCo_2_S_4_

**Figure 2:**
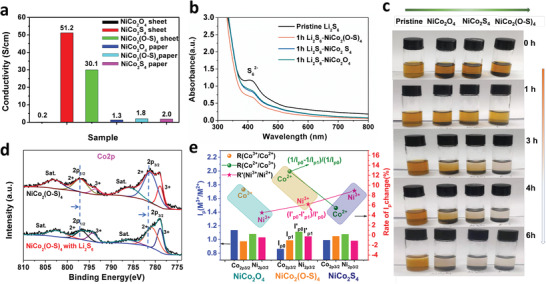
a) Conductivities of NiCo_2_O_4_, NiCo_2_(O–S)_4_, NiCo_2_S_4_ sheets and supported MWCNT papers. b) UV‐vis spectra of 0.1mM Li_2_S_6_ solution before and after 1 h absorption. c) Optical images of NiCo_2_O_4_, NiCo_2_(O–S)_4_ and NiCo_2_S_4_ soaked in the 10 mM Li_2_S_6_ solution. d) High‐resolution XPS spectra of Co 2p for NiCo_2_(O–S)_4_ with and without Li_2_S_6_ catholyte adsorption. e) The ratio of M^3+^/M^2+^ 2p_3/2_ peak intensity (*I*_p_) and the rate of *I*_p_ change.

We hypothesized that high‐density oxygen doping could make NiCo_2_(O–S)_4_ (similar to S2‐NiCo_2_(O–S)_4_) less conducting than natively oxidized NiCo_2_S_4_ but increase its affinity to LiPS owing to the introduction of more Li‐O interactions for trapping S*_n_* ^2–^. To demonstrate this, the electronic conductivity measurements and LiPS adsorption tests were carried out with four‐point probe measurements and UV‐vis spectroscopy, respectively. As shown in **Figure** **2**a, the conductivities of the natively oxidized NiCo_2_S_4_ and NiCo_2_(O–S)_4_ sheets can reach 51.2 and 30.1 S cm^–1^, respectively, while NiCo_2_O_4_ only showed a conductivity of 0.3S cm^–1^. Composite papers containing multiwall carbon nanotubes (MWCNTs) and cellulose nanofiber (CNF) mixed with NiCo_2_O_4_, NiCo_2_(O‐S)_4,_ and NiCo_2_S_4_ were also prepared for further investigation as LiPS cathodes as described in the experimental section. The conductivity order of these composite papers was NiCo_2_S_4_ (2.0 S cm^–1^), NiCo_2_(O–S)_4_ (1.8 S cm^–1^), and NiCo_2_O_4_ (1.3 S cm^–1^), which was just consistent with reasonable estimation.

To test the difference in LiPS interactions, adsorption tests were carried out by adding 15 mg of each of the materials into 2 mL vials containing 0.1 mM Li_2_S_6_‐DME/DOL solution. As shown in Figure 2b, the absorbance of the S_4_^6–^ peak drops to much lower intensities after the addition of the NiCo_2_(O–S)_4_ sample, indicating its adsorbed more LiPS (had stronger interactions with LiPS) than NiCo_2_O_4_ and NiCo_2_S_4_, which displayed similar S_4_^6–^ absorbance. Further time‐dependence of the adsorption tests were investigated; as shown by the photographs in Figure 2c and Figure S12, Supporting Information, NiCo_2_(O–S)_4_ and S2‐NiCo_2_(O–S)_4_ show the improved adsorption of LiPS compared to pristine NiCo_2_S_4_ and NiCo_2_O_4_. The chemical interactions between the LiPS and these adsorbents were further investigated via XPS. The spectra of NiCo_2_O_4_, NiCo_2_(O–S)_4,_ and NiCo_2_S_4_ with and without Li_2_S_6_ retrieved from the adsorption test solutions were analyzed. Since all these samples exhibit Lewis acid‐base bonding (*M*–S) with LiPS, the peaks of Ni 2p and Co 2p all show a shift to lower binding energies after interacting with Li_2_S_6_ as shown in Figure 2d, Figure S13–S15, Supporting Information.^[^ ^1^, ^19^ ^]^ It is hard to identify and compare the shifts in the binding energies among these samples because they are quite similar and it seems that each one has a unique preferred interaction site with Li_2_S_6_. By comparing these fitted Co 2p and Ni 2p peaks, it is interesting to see obvious intensity changes for the fitted metal (M^2+^ and M^3+^, *M* = Co or Ni) peaks with and without Li_2_S_6_ interaction. For example, the intensities of the Co^2+^ peak in NiCo_2_(O–S)_4_ and NiCo_2_S_4_ decrease more than the Co^3+^ peak after interacting with Li_2_S_6_ (Figure 2d, Figure S14, Supporting Information), which suggests that the Co^2+^ site dominates the LiPS adsorption in the Co^3+^‐Co^2+^ couple for NiCo_2_(O‐S)_4_ and NiCo_2_S_4_. Meanwhile, the spectra show that the intensities of the M^3+^ peaks for NiCo_2_O_4_, NiCo_2_(O‐S)_4_ and NiCo_2_S_4_ decrease more obviously than M^2+^ after interacting with Li_2_S_6_, suggesting that M^3+^ sites may also dominate the LiPS adsorption depending on the sample and M^2+^‐ M^3+^ couple. Thus, we use the ratio of the M^3+^ to M^2+^ 2p_3/2_ peak intensity, which is marked as *I*_p_ (M^3+^/ M^2+^) in Figure 2e, to clarify the preferred LiPS interaction sites. The columns in Figure 2e show that, for NiCo_2_(O‐S)_4_ and NiCo_2_S_4_, I_p_ (Co^3+^/ Co^2+^) increases while I_p_ (Ni^3+^/ Ni^2+^) decrease after interacting with Li_2_S_6_, suggesting that NiCo_2_(O–S)_4_ and NiCo_2_S_4_ share similar preferred interacting sites (Co^2+^ and Ni^3+^) with LiPS. On the other hand, the Co^3+^ and Ni^3+^ are the sites that interact with LiPS in NiCo_2_O_4_. The *I*_p0_ and *I*_p1_ represent the intensity of the fitted Co_2p3/2_ peaks without and with Li_2_S_6_ absorption, respectively, while *I*’_p0_ and *I*’_p1_ are associated with Ni_2p3/2_. Since NiCo_2_(O–S)_4_ and NiCo_2_S_4_ share similar preferred interacting sites (Co^2+^ and Ni^3+^) with LiPS, the rates of *I*_p_ change (R(Co^3+^/Co^2+^) = (*I*_p1_ − *I*_p0_)/*I*_p1_; R(Ni^3+^/Ni^2+^) = (*I*’_p0_ − I’_p1_)/*I*’_p0_ were also calculated to roughly compare the dominated LiPS interaction sites of Co^2+^ and Ni^3+^. Specifically, NiCo_2_O_4_ is Co^3+^ and Ni^3+^ dominated, R(Co^3+^/Co^2+^) for NiCo_2_O_4_ here is calculated by (*I*_p0_ − *I*_p1_)/*I*_p0_ and R(Ni^3+^/Ni^2+^) is similarly calculated for NiCo_2_(O–S)_4_ and NiCo_2_S_4_. As shown in Figure 2e, the R(Co^3+^/Co^2+^) of NiCo_2_O_4_ and NiCo_2_(O–S)_4_ are much higher than NiCo_2_S_4_ while R(Ni^3+^/Ni^2+^) increases from 4.5, 6.2 to 8.9% as NiCo_2_O_4_ gradually changed to be NiCo_2_(O–S)_4_ and NiCo_2_S_4_. This indicates that the Co dominates the Li_2_S_6_ interaction site of the oxide and oxide‐sulfides, but Ni dominates the Li_2_S_6_ interaction site of the sulfide. NiCo_2_(O–S)_4_ shares the similar Co^2+^ preferred interaction site with NiCo_2_S_4_, but as high‐density O is introduced, the increased R(Co^3+^/Co^2+^) suggests that the Co^2+^ site of NiCo_2_(O–S)_4_ is greatly activated and enhanced without too much decrease in Ni^2+^ site, which can be an explanation for its better absorption properties compared to NiCo_2_S_4_ and NiCo_2_O_4_. Figure S16, Supporting Information, also shows the fitted O 1s spectra of NiCo_2_O_4_ and NiCo_2_(O–S)_4_ with and without adsorption of Li_2_S_6_. The fitted O1 peak centered near 529.5 eV represents the *M*–O bond of the samples. After interacting with Li_2_S_6_, the O1 peaks decrease in intensity in both samples, which can be attributed to the formation of Li–O–M species, suggesting that there is electron transfer from the LiPS to the O atoms. Using the same method, we can also obtain the R(O_2_/O_1_) data, in which NiCo_2_O_4_ shows R of 78.1% while NiCo_2_(O–S)_4_ shows 60.0%, indicating that our proposed Li–O interaction is greatly introduced to NiCo_2_(O‐S)_4._ It should be emphasized that the interactions between LiPS and the samples are complicated and we cannot rely completely on the R(M^3+^/M^2+^) or R(O_2_/O_1_) to quantify the differences in LiPS absorption ability difference, especially between NiCo_2_O_4_ and NiCo_2_(O–S)_4_ because they show different Li_2_S_6_ interaction sites (Co^3+^‐Ni^3+^ for NiCo_2_O_4_, Co^2+^‐Ni^3+^for NiCo_2_(O–S)_4_). However, we suspect that the Co^2+^ site can be more effective than Co^3+^ for absorbing LiPS, which can explain why NiCo_2_(O–S)_4_ shows enhanced LiPS absorption ability compared to NiCo_2_O_4_ during the adsorption and UV‐vis spectroscopy tests. Moreover, to simply understand the O‐doping effect on LiPS absorption for O‐NiCo_2_S_4_ and pure NiCo_2_S_4_, DFT is also adopted to simulate the adsorption energies (*E*_ads_) with Li_2_S*_n_* (*n* = 1, 4, 6, 8) and charge density surrounding the adsorption sites. As shown and discussed in Figure S17, Supporting Information, the adsorption energies on the O‐NiCo_2_S_4_ (311) surface are more negative than those for the pure NiCo_2_S_4_ (311) surface and the charges are especially aggregated around the oxygen atoms, indicating enhanced interaction of lithium sulfides on the oxygen sites of O‐NiCo_2_S_4_ (311) surface. Bader charge analysis can quantitatively describe charge transfer between adsorbates and substrates. It shows in Table S2, Supporting Information that electrons are transferred from the Li in the LiPS to the S/O atoms, with more electrons transferred to O‐NiCo_2_S_4_ compared to NiCo_2_S_4_, which is consistent with the higher Pauli electronegativity of O compared to S. Interestingly, compared with the S atoms at the pure NiCo_2_S_4_ (311) surface, the substituting O atom gains more electrons (over 3 times). The excess electrons going to the O atoms not only come from Li_2_S*_n_*, but also are donated by neighboring atoms. Hence, the charge density surrounding the adsorption sites is redistributed to modulate the adsorption behavior of Li‐S species, which may be beneficial for lithium–sulfur redox.

### 2.3 Redox and Deposition Kinetics of S_8_–Li_2_S Assisted with NiCo_2_O_4_, NiCo_2_(O–S)_4_ and NiCo_2_S_4_ Papers

**Figure 3:**
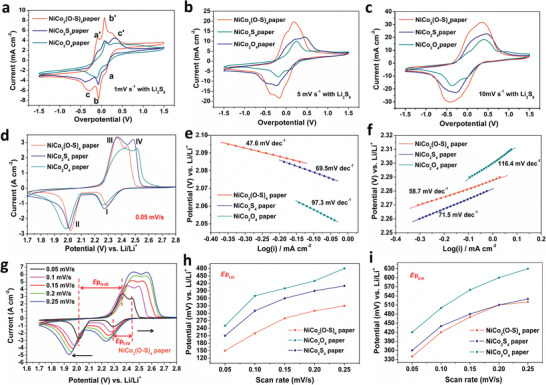
a) CV curves of the symmetric cells at scan rates of 1 mV s^–1^, b) 5 mV s^–1^ and c) 10 mV s^–1^ with NiCo_2_O_4_, NiCo_2_(O–S)_4_ and NiCo_2_S_4_ papers as the electrodes. The electrolyte was 0.5 M Li_2_S_6_ in DOL/DME (1:1 in volume). d) CV curves of the Li–S full cells at a scan rate of 0.05 mV s^–1^. e) Tafel plots were calculated from the CV curves for the low plateau reduction peak at around 2.0 V and f) the first oxidation peak at around 2.35 V. g) CV curves of NiCo_2_(O–S)_4_ paper supported Li–S full cells from scan rates of 0.05 to 0.25 mV s^–1^. Corresponding peak potential difference of *E*p_I‐IV_ h) and *E*p_II‐III_ i).

The low conductivities of S_8_ and Li_2_S_2_/Li_2_S, and the solubility of LiPS in the electrolyte greatly limit the conversion kinetics and utilization of S_8_; thus, materials with both high electronic conductivity and LiPS affinity are more attractive for fabricating sulfur cathodes.^[^ ^20^ ^]^ High‐density O doped NiCo_2_(O‐S)_4_ shows more LiPS affinity than NiCo_2_O_4_ and NiCo_2_S_4_ and it is also as conducting as NiCo_2_S_4_. Thus, it can be a great candidate for catalyzing the S_8_‐Li_2_S redox reaction. To investigate this, free‐standing NiCo_2_O_4_, NiCo_2_(O–S)_4_ and NiCo_2_S_4_ papers were prepared and implemented as current collectors for the S_8_‐LiPS‐Li_2_S conversion reaction as described in the experimental section. The characteristic peaks of the MWCNTs, NiCo_2_O_4_, NiCo_2_(O‐S)_4_ and NiCo_2_S_4_ were clearly observed in the XRD diffraction patterns (Figure S18, Supporting Information), suggesting no significant phase change in our preparation process. To first investigate the catalytic ability of these samples, symmetric cells were assembled with two identical papers as electrodes in 40 µL 0.5 mol L^–1^ Li_2_S_6_ as described in the experimental section. Cyclic voltammetry (CV) tests were conducted in the voltage range −1.5 ‐ 1.5 V at 1 mV s^–1^ (**Figure** **3**a), 5 mV s^–1^ (Figure 3b), and 10 mV s^–1^(Figure 3c). The capacitive current response and the small peak separation between the reduction and oxidation peaks both demonstrate the fast and reversible conversion of LiPSs.^[^ ^21^ ^]^ When comparing the capacitive current response from 1 to 10 mV s^–1^, it is obvious that the NiCo_2_(O–S)_4_ paper exhibits much higher current response than that of symmetrical cells with NiCo_2_O_4_ and NiCo_2_S_4_ papers. Figure 3a also shows the three distinct pairs of redox peaks for all samples, where the reduction peaks labelled a, b and c are attributed to the reduction of S_8_ to Li_2_S_6_, Li_2_S_6_ to Li_2_S_4_ and Li_2_S_4_ to Li_2_S_2_/Li_2_S, respectively. The oxidation peaks a’, b’ and c’ are associated with oxidation of Li_2_S_2_/Li_2_S to Li_2_S_4_, Li_2_S_4_ to Li_2_S_6_ and Li_2_S_6_ to S_8_. Note that the cell with the NiCo_2_(O‐S)_4_ paper exhibits a much smaller peak separation than NiCo_2_O_4_ and NiCo_2_S_4_ papers at all scan rates, confirming that NiCo_2_(O‐S)_4_ significantly enhances the kinetics of the lithiation/delithiation reactions for polysulfides conversion. Meanwhile, the smaller current response and bigger peak separation of NiCo_2_O_4_ paper should be ascribed to its high charge transfer barrier because of its low conductivity. The weaker interactions between NiCo_2_S_4_ with the LiPS contribute to the more sluggish kinetics for LiPS conversion compared to the NiCo_2_(O‐S)_4_ paper.

The different catalyzing effects of the materials can also be confirmed by the electrochemical impedance spectra (EIS) in Figure S19, which show that the symmetric cell with NiCo_2_(O–S)_4_ paper electrodes has a much smaller semicircle (associated with the charge transfer resistance, *R*_ct_) than those with NiCo_2_O_4_ or NiCo_2_S_4_ electrodes, indicating that NiCo_2_(O–S)_4_ is more effective for enhancing the charge transfer for LiPSs conversion. CV tests were used to further verify the ability of NiCo_2_(O–S)_4_ for enhancing the kinetics in Li–S cells. As shown in Figure 3d, the reduction peaks I and II represent the reduction of S_8_ to LiPS and LiPS to Li_2_S_2_/Li_2_S while the oxidation peaks of III and IV are associated with oxidation of Li_2_S_2_/Li_2_S to LiPS and LiPS to S_8_, respectively. The much higher potential of the reduction peaks and lower potential of oxidation peaks, which are clearly split, suggests that the NiCo_2_(O–S)_4_ paper promoted the conversion between S_8_, soluble LiPS, and Li_2_S_2_/Li_2_S.^[^ ^22^ ^]^ The catalytic effects of these three materials were also compared by calculating the Tafel plots of the reduction (I and II) and oxidation (III) peaks in Figure 3d. As shown in Figure 3e,f, and Figure S20, Supporting Information, for both the reduction and oxidation processes, the fitted slopes clearly follow the order of NiCo_2_(O–S)_4_ < NiCo_2_S_4_ < NiCo_2_O_4_, which suggests that the high‐density O‐doped NiCo_2_(O‐S)_4_ has a better catalytic activity for the fast reduction and oxidation of S_8_, LiPSs, and Li_2_S_2_/Li_2_S while the catalytic activity of NiCo_2_O_4_ is relatively sluggish because of its much lower conductivity. Further, CV curves of the Li–S cells with NiCo_2_(O–S)_4_, NiCo_2_O_4,_ and NiCo_2_S_4_ papers were also recorded at various scan rates from 0.05‐0.25 mV s^−1^ as shown in Figure 3g and Figure S21–S22, Supporting Information. The peak potential differences of I–IV (*E*p_I‐IV_) and II–III (*E*p_II‐III_) redox couples were also used for tracking the changes upon going to higher scan rates. As shown in Figure 3h,i, we can clearly verify that the cell with the NiCo_2_(O–S)_4_ paper has lowest *E*p_I–IV_ and *E*p_II–III_ at all scan rates, and its slopes between two adjacent scan rates seems to gradually overlap with NiCo_2_S_4_, suggesting the more conducting NiCo_2_(O–S)_4_ can reduce the charge transfer barrier similarly as NiCo_2_S_4_. In contrast, NiCo_2_O_4_ suffers from its low conductivity and the cell shows the highest *E*p_I–IV_, *E*p_II–III_ and much higher slopes between two scan rates. These results all demonstrate the enhanced polysulfide conversion kinetics in the entire charge/discharge processes for the NiCo_2_(O–S)_4_ assisted Li–S cell.

**Figure 4:**
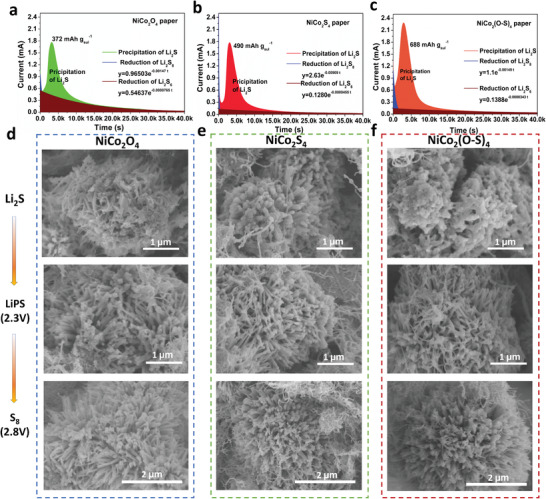
a) Li_2_S nucleation curves of NiCo_2_O_4_ paper, b) NiCo_2_S_4_ paper and c) NiCo_2_(O–S)_4_ paper. Corresponding morphologies electrodes after Li_2_S nucleation, dissolution to LiPS, and deposition of S_8_ on surfaces of d) NiCo_2_O_4_, e) NiCo_2_S_4_, and f) NiCo_2_(O–S)_4_.

Apart from enhancing polysulfide redox kinetics as mentioned above, we supposed that NiCo_2_(O–S)_4_ would also show better catalyzing effects for Li_2_S nucleation because of its sufficient conductivity and high affinity to LiPS. To prove this, Li_2_S precipitation experiments were carried out by using the potentiostatic discharge method.^[^ ^23^ ^]^ According to our previous research and other reports,^[^ ^20a^ ^,^ ^24^ ^]^ when LiPS is discharging to form solid Li_2_S_2_/Li_2_S, driven by the so‐called “local concentration effect”, Li_2_S_2_/Li_2_S prefers to precipitate and gradually grow to form thick coatings on these “dual high” (high conductivity and high LiPS affinity) materials. Thus, the easier the Li_2_S_2_/Li_2_S precipitation and thicker the coating, the faster the deposition kinetics and higher sulfur utilization that can be achieved by the LiPS conversion catalysts such as the NiCo_2_(O–S)_4_, NiCo_2_O_4_ and NiCo_2_S_4_. Comparing the Li_2_S nucleation curves shown in **Figure** **4**a,c, we can clearly see that the capacity of precipitated Li_2_S on NiCo_2_(O–S)_4_ paper is about 688 mAh g_s_^–1^, which is much higher than those on NiCo_2_S_4_ paper (490 mAh g_s_^–1^) and NiCo_2_O_4_ paper (372 mAh g_s_^–1^). Besides, the Li–S cell with NiCo_2_(O–S)_4_ paper exhibited the highest peak current while NiCo_2_S_4_ and NiCo_2_O_4_ share a similar peak current, suggesting faster LiPSs trapping and nucleation of Li_2_S on NiCo_2_(O–S)_4_ paper. The pristine electrodes and deposited morphology of Li_2_S in precipitation tests were also characterized by SEM as shown in Figure S23, Supporting Information, and Figure 4d–f, respectively. The pristine electrodes contain flower‐like particles which are physically mixed with MWCNT (Figure S23, Supporting Information). After constant potential depositing Li_2_S, Figure 4d shows a little bit of Li_2_S solid deposits on the 1D nanofibers of the NiCo_2_O_4_ particle; while for NiCo_2_S_4_ and NiCo_2_(O–S)_4_ (Figure 4e,f), thick Li_2_S coating is clearly deposited on the 1D nanofibers and the surface of NiCo_2_(O–S)_4_ (Figure 4f) particle is especially rougher than NiCo_2_S_4_, in which Li_2_S deposits almost wrapped the 1D nanofibers. These morphologies verify that with sufficient electronic conductivity (as high as NiCo_2_S_4_) and superior interaction with LiPS, NiCo_2_(O–S)_4_ is more powerful for accelerate Li_2_S nucleating and growing to be thicker, thus the high capacity of sulfur can be achieved. On the other hand, the oxidation morphology of deposited Li_2_S was further investigated by using a normal galvanostatic charging process as marked in Figure 4d–f. When the assembled cells are charged to 2.3V, we can see that the deposited Li_2_S are gradually dissolved and NiCo_2_O_4_, NiCo_2_S_4_ and NiCo_2_(O‐S)_4_ share its similar morphology. However, when they are charged to 2.8V, some solid participants can be detected on the surface of NiCo_2_S_4_ and NiCo_2_(O–S)_4_ while the surface of NiCo_2_O_4_ particles remains smooth and clean. Besides, the 1D nanofibers of NiCo_2_(O–S)_4_ particle becomes much rougher than those of NiCo_2_S_4_, indicating the easier dissolution of Li_2_S and deposition of S_8_ on NiCo_2_(O–S)_4_ surface than that on NiCo_2_O_4_ and NiCo_2_S_4_. Noting that these SEM images were taken just for 1 cycled electrode, much details for long cycled electrodes below would show more difference for the different materials. Anyway, these results clearly confirm that with the help of high‐density O doping, NiCo_2_(O‐S)_4_ facilitates the effective deposition of both Li_2_S and S_8_, suggesting to be a desired catalyst for improving sulfur utilization in high areal capacity (>3mAh cm^–2^) Li–S cells.

### 2.4 Electrochemical Performance of Corresponding Li–S Cells

**Figure 5:**
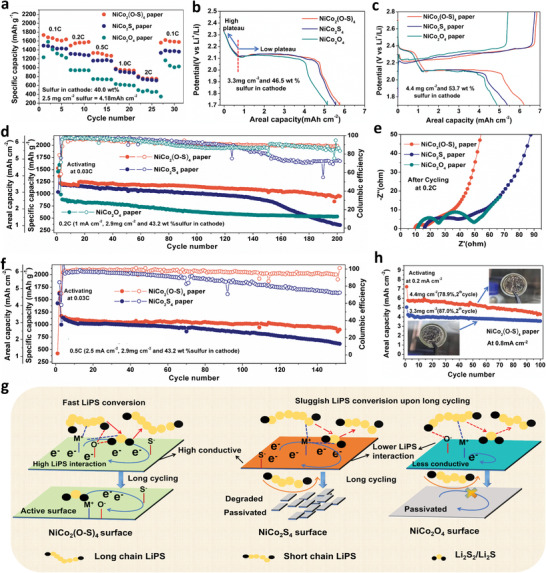
a) Rate performance of different paper supported Li–S cells from 0.1C to 2C with sulfur loading of 2.5 mg cm^–2^. b) The first discharge curves of NiCo_2_O_4_, NiCo_2_S_4_ and NiCo_2_(O–S)_4_ paper supported Li_2_S_8_ catholyte with sulfur loading of 3.3 mg cm^–2^, c) and the corresponding 3^rd^ discharge‐charge curves at 0.16 mA cm^–2^. d) Cycling performance of NiCo_2_O_4_, NiCo_2_S_4_ and NiCo_2_(O–S)_4_ paper supported Li–S cells with sulfur loading of 2.9 mg cm^–2^ at 0.2C, e) and corresponding EIS spectra after cycling. f) Cycling performance of NiCo_2_O_4_, NiCo_2_S_4_ and NiCo_2_(O–S)_4_ paper supported Li–S cells with sulfur loading of 2.9 mg cm^–2^ at 0.5C. g) Schematic of the long‐term cycling of the different catalyzing surfaces for LiPS conversion. h) Cycling performance of NiCo_2_(O–S)_4_ paper supported Li–S cells with sulfur loading of 3.3 and 4.4 mg cm^–2^ at 0.8 mA cm^–2^. Noting that the paper electrodes comprising the Ni‐Co compounds, as well as the small quantity of Li_2_S_6_ additives in the electrolyte can contribute extra capacities mainly during the activating cycles.

To compare the effect of the differences in catalyzing performance of these NiCo compounds in the cycling behavior of the Li–S cells, coin cells were assembled using lithium metal as anode and NiCo_2_O_4_, NiCo_2_S_4_ and NiCo_2_(O–S)_4_ papers loaded with Li_2_S_8_ solution as cathode. Different amounts of Li_2_S_8_ were loaded corresponding to sulfur loading from 2.5, 2.9, 3.3 to 4.4 and 8.75 mg cm^–2^ for >3 mAh cm^–2^ Li–S cells. **Figure** **5**a first shows the rate performance of the cells with a low sulfur loading of 2.5 mg cm^–2^ (40.0 wt.% in cathode), which confirms that the NiCo_2_(O–S)_4_ paper outperforms the other two at all rates. The corresponding discharge‐charge curves in Figure S24, Supporting Information further show that the NiCo_2_(O–S)_4_ supported cell especially has a higher initial capacity at the higher voltage plateau (from S_8_–Li_2_S_4_ redox), suggesting its better catalyzing effect for converting S_8_ and high‐chain LiPS compared to NiCo_2_S_4_ and NiCo_2_O_4_. Specifically, the cell with the NiCo_2_(O–S)_4_ paper can deliver a high capacity of about 1268 mAh g^–1^ at 0.5C, which is equal to a sulfur utilization of 75.8% and areal capacity of 3.2 mAh cm^–2^. The cycling performance of the NiCo_2_(O–S)_4_ paper supported cell at 1C is also shown in Figure S25, showing high sulfur utilization of 50.8% and a high areal capacity of 2.1 mAh cm^–2^ after 230 cycles. The improved LiPS–Li_2_S conversion by NiCo_2_(O–S)_4_ is also verified from the activating discharge and charge curves for even high sulfur loading (3.3 mg cm^–2^ and 4.4 mg cm^–2^) cells as shown in Figure 5b,c. As we applied a quite low current density (0.16 mA cm^–2^) for achieving full conversion of S_8_ or high‐chain LiPS to Li_2_S_4_ at the higher voltage plateau, the capacity of the lower plateau would demonstrate the catalyzing ability of different samples for Li_2_S_4_–Li_2_S conversion. Figure 5b exactly shows that though the three cells share a similar initial activated capacity of LiPS–Li_2_S_4_ (0.83 mAh cm^–2^), the cells with more conducting NiCo_2_(O–S)_4_ and NiCo_2_S_4_ clearly have higher capacities associated with the low voltage plateau than the NiCo_2_O_4_ paper. When cells with even higher sulfur loading were further activated at the 2^nd^ cycle as shown in Figure 5c, the result confirms that NiCo_2_(O–S)_4_ still outperforms NiCo_2_S_4_ and NiCo_2_O_4_ for both S_8_‐Li_2_S_4_ and Li_2_S_4_–Li_2_S conversion. It should be noted that the papers comprising the Ni–Co compounds, as well as the small quantity of Li_2_S_6_ additives in the electrolyte can contribute extra capacities mainly during the activating cycles (Figure S26–S28, Supporting Information), thus the areal capacities of the first cycles in Figure 5b,h exceed the theoretical value. Long‐term cycling tests of these cells with a sulfur loading of 2.9 mg cm^–2^ (43.2 wt.% in cathode) were also carried out to check the stability of these electrodes. As shown in Figure 5d–f, the NiCo_2_(O–S)_4_ paper supported cell can realize a capacity of 962 mAh g^–1^ (2.8 mAh cm^–2^) at 0.2C after 200 cycles and 922 mAh g^–1^ (2.7 mAh cm^–2^) at 0.5C after 150 cycles, while NiCo_2_O_4_ paper shows lowest capacity and NiCo_2_S_4_ paper suffers rapid capacity fading in the longer cycles. The coulombic efficiency of the NiCo_2_S_4_ paper supported cells also decreases faster during long‐term cycling, suggesting its worse stability than NiCo_2_(O‐S)_4_ paper. EIS spectra after cycling at 0.2C (Figure 5e) further show the cell with NiCo_2_(O–S)_4_ paper has the smallest charge transfer resistance, which is attributed to the stable exposed catalytic surfaces during cycling.

Optical images after cycling (Figure S29, Supporting Information) show less damage to the surfaces of lithium anodes taken from cells with NiCo_2_(O–S)_4_ paper compared to the other two, suggesting more suppression of the LiPS shuttle because of strong LiPS trapping and conversion on the NiCo_2_(O–S)_4_ surface. The morphologies of the cycled electrodes at 0.2C were also investigated by SEM to characterize the surface and different parts of the electrode cross‐section (Figure S30, Supporting Information). Previous research has suggested that the continuous precipitation and dissolution of S_8_–LiPS–Li_2_S during cycling results in sulfur redistribution towards the electrode surface; thus, high concentrations of LiPS gradually aggregate near the electrode surface, leading to sluggish kinetics for LiPS conversion, deposition of inactive sulfide in every cycle, and formation of an insulating passivated layer that will block the electrode active sites.^[^ ^25^ ^]^ A good catalyst would greatly alleviate the passivation process and retain a stable catalyzing surface for long‐term cycling.^[^ ^26^ ^]^ The particle morphologies on the surface of the electrode in Figure S30a, Supporting Information, clearly confirms that NiCo_2_(O–S)_4_ retains a stable particle shape with obvious 1D nanofibers covered by some precipitates, which suggests there is a higher active exposed surface that is able to catalyze the LiPS conversion. In contrast, the NiCo_2_O_4_ and NiCo_2_S_4_ particles seem to suffer from structural changes that cause the 1D nanofibers to be decomposed and become covered with thick precipitates, which suggests the surfaces have been inactivated. The top cross‐section (closer to the electrode surface) in Figure S30b, Supporting Information, demonstrates that NiCo_2_(O–S)_4_ and NiCo_2_S_4_ electrodes are still sufficiently porous for LiPS diffusion, but it might be blocked in the NiCo_2_O_4_ electrodes. Going deeper to the middle cross‐section, we can see in Figure S30c, Supporting Information (closer to top cross‐section) that NiCo_2_(O–S)_4_ particles still show stable flower‐like morphology with clearly‐visible 1D nanofibers that were uniformly covered with sulfide precipitation as usual. However, the flower‐like NiCo_2_S_4_ particle is decomposed to form aggregated, bulk precipitates that are less conductive for SEM imaging; while the NiCo_2_O_4_ particle is wrapped by thick precipitates that completely block the active catalyzing surface. Figure S30d,e, Supporting Information, further show the deeper part of the electrode (close to positive coin cell cases), which verifies that NiCo_2_(O‐S)_4_ is chemically stable during long‐term cycling and can keep an active surface, but NiCo_2_S_4_ is chemically unstable and thus suffering from degradation. Owing to its low conductivity, it is much easier for NiCo_2_O_4_ to have inactive sulfide precipitation completely block its active surface. These SEM results clearly demonstrate the failure mechanism of NiCo_2_O_4_ and NiCo_2_S_4_ particles for LiPS conversion upon long‐term cycling. As illustrated in Figure 5g, our results suggest that NiCo_2_(O–S)_4_ not only has high conductivity and high LiPS affinity, but also more chemical stability upon long‐term LiPS conversion. This is all attributed to the large amount of O doping that greatly prevents the passivation of the catalyzing surface upon Li_2_S_2_/Li_2_S deposition and dissolution.

The NiCo_2_(O‐S)_4_ papers were also used for fabricating higher areal capacity Li–S cells with high sulfur utilization. The cycling performance for the 3.3 mg cm^–2^ sulfur loading (46.5 wt.% in cathode) cell (Figure 5h) can achieve an areal capacity of 4.205 mAh cm^–2^ at the beginning and 3.565 mAh cm^–2^ (sulfur utilization of 73.0%) after 100 cycles. By increasing the sulfur loading to 4.4 mg cm^–2^ and also using a 7.1 mg cm^–2^ NiCo_2_(O–S)_4_ paper (40.0 wt.% in cathode), an areal capacity of 5.77 mAh cm^–2^ at the beginning and 4.28 mAh cm^–2^ (sulfur utilization of 58.5%) after 100 cycles can be realized. The inset in Figure 5h shows the dissembled lithium anodes, which are damaged due to the high capacity of Li deposition and stripping. Though the long‐term cycling performance of these cells cannot be achieved because of the lithium anode, these results clearly demonstrate that the high‐density O‐doped NiCo_2_(O–S)_4_ greatly outperforms the oxide and sulfides for improving the redox kinetics of Li–S cells. There is also potential for applying this high‐density O‐doping chemistry for other metal compounds to develop more active and stable catalysts for improving the performance of Li–S cells.

**Figure 6:**
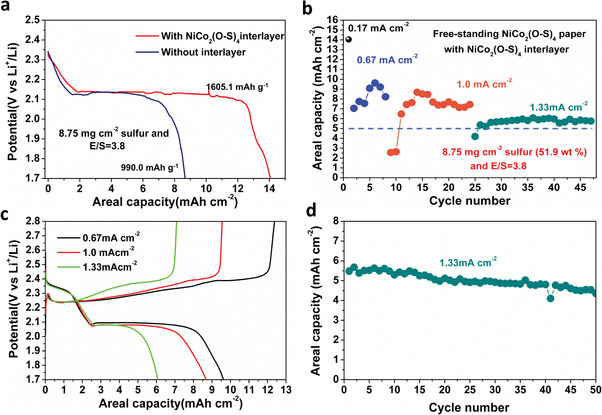
a) The first activating curves of NiCo_2_(O–S)_4_ paper supported Li_2_S_8_ catholyte with and without interlayer. b) Rate performance of NiCo_2_(O–S)_4_ paper and interlayer assisted Li–S cell with lean electrolyte and high sulfur loading. c) Rate discharge‐charge curves at the highest of the capacity cycle. Long cycling performance at 1.33 mA cm^–2^.

The good effects of high‐density O‐doped NiCo_2_(O–S)_4_ for improving the redox kinetics of Li–S cells are also demonstrated in high sulfur loading cathode with lean electrolyte. In order to further enhance the trapping of LiPS, a NiCo_2_(O–S)_4_ interlayer was also applied as described in supporting information. **Figure** **6**a shows the improved areal capacity for NiCo_2_(O–S)_4_ interlayer assisted cells with a sulfur loading of 8.75 mg cm^–2^ and E/S of 3.8 µL g^–1^. An initial areal capacity of 14.05 mAh cm^–2^ was achieved at 0.17 mA cm^–2^ in cells with the interlayer. The rate capacity curve is plotted by merging the initial cycles at 0.17 ‐ 1.33 mA cm^–2^ in Figure 6b, indicating these areal capacities can be all above 5 mAh cm^–2^ and the highest capacity at 1 mA cm^–2^ can be 8.68 mAh cm^–2^_._ The capacity after 16 cycles at 1.0 mA cm^–2^ is as high as 7.43 mAh cm^–2^. The discharge‐charge curves at different rates and long‐term cycling performance at 1.33 mA cm^–2^ are further shown in Figure 6c,d. The discharge profile in Figure 6c still exhibits an intact high voltage plateau at 2.3 V and a low voltage plateau at 2.08 V. In addition, the total capacity is still more than 3 times higher than the high voltage plateau capacity (the theoretical ratio is 4) at 0.67 and 1 mA cm^–2^, owing to the enhanced electrochemical kinetics within the cell. Figure 6d shows the cycling performance at 1.33 mA cm^–2^. After 50 cycles, a capacity of 4.54 mAh cm^–2^ was achieved. Taking into account the high areal capacity, lean electrolyte, and the free‐standing character of the paper electrodes, our work can be competitive with these recently reported works (Table S4, Supporting Information).^[^ ^20^, ^26^, ^28^ ^]^ Since our freestanding papers are free of Al foil and use high sulfur loading, they can be used in pouch cells instead of the double‐sided S coated Al foil. In that case, the self‐standing paper with high sulfur loading of 8.75 mg cm^–2^ would even perform better and lead to higher energy density and utilization of sulfur.

Furthermore, we also applied this high‐density O‐doping approach to other compounds with high conductivity and find that this simple method is able to obtain higher oxygen content in Mo–OS, Ni–OS, Ni–OP, and Co–OS than the natively oxidized MoS_2_, NiS, Ni_2_P, and Co_3_S_4_. As shown in Figure S31–S39, Supporting Information, these materials had EDS spectra that showed higher O:S or O:P atom ratios and better LiPS adsorption than natively oxidized sulfides or phosphides. XRD patterns of Ni–OS, Ni–OP and Co–OS show mixed oxides or sulfide phases while Mo–OS shows O‐doped states of MoS_2_ which is similar to NiCo_2_(O–S)_4_. Though in some cases (e.g., for Ni–OS and Ni–OP) the oxide (i.e., NiO) displays the best LiPS adsorption, oxides are usually not as conductive as sulfides and may not result in high utilization of sulfur or resistance to passivation upon cycling, similar to NiCo_2_O_4_. Noting that high‐density O‐doping is able to maintain the conductivity, improve the LiPS absorption ability and chemical stability of the original sulfides, this approach can be a promising method for preparing more high‐performance sulfur hosts. Moreover, it should be also emphasized that NiCo_2_(O‐S)_4_ and Mo‐OS are special cases because they follow the structure of their sulfides and are not a simple mixed composites of oxides and sulfides like the other Ni–OS, Ni–OP, and Co–OS materials, as indicated by the XRD patterns. Overall, our work demonstrates a simple way for doping large amounts of oxygen into sulfide compounds, the special catalyst NiCo_2_(O–S)_4_ or Mo–OS, and a series of potential materials for high performance of sulfur cathodes. The unique NiCo_2_(O–S)_4_ we prepared can also have potential uses in other research areas.

## 3 Conclusions

To the best of our knowledge, this is the first study that uses a high amount of oxygen doped bimetal sulfide for improving the redox kinetics of Li‐S batteries. By simply controlling the reaction time and quantity of the sulfidation reactant, we find the possibility of preparing a high amount oxygen‐doped NiCo_2_S_4_ which we called NiCo_2_(O–S)_4._ We demonstrated NiCo_2_(O–S)_4_ greatly outperformed the NiCo oxides and natively oxidized sulfides for trapping LiPS and catalyzing LiPS conversion. Taking the advantages of chemical stability, high conductivity, enhanced polar‐polar interaction of Li–O–Co (Ni) species, and activated Co (Ni) facets for absorbing S*_n_* ^2–^, the as‐made cell with free‐standing NiCo_2_(O–S)_4_ paper greatly showed improved potential for fabricating high areal capacity Li–S batteries. By further applying self‐standing NiCo_2_(O–S)_4_ paper and its assisted interlayer for lean electrolyte Li‐S cells, we achieve the highest areal capacity of 8.68 mAh cm^–2^ at 1mA cm^–2^ even with 8.75 mg cm^–2^ sulfur loading and low E/S of 3.8 ul g^–1^. There is also potential for applying this high‐density O‐doping chemistry for preparing other promising metal compounds or composites. We expect that this study will give more impetus for exploring the highly conductive, chemically stable, and high LiPS affinity metal compounds with high‐density O‐doping for high‐performance Li–S batteries.

## Conflict of Interest

The authors declare no conflict of interest.

## Author Contributions

H.W. conceptualized the work, and assisted in data curation, formal analysis, funding acquisition, methodology, visualization, project administration, resources, supervision, visualization, writing draft‐reviewing and editing. Y.L. did the conceptualization, data curation, formal analysis, methodology, investigation, validation, software, visualization, writing draft‐reviewing and editing. D.W. conducted investigation, data curation, software, visualization (DFT calculation). H.W. and Y.G. did the validation and review. H.C. performed data curation, formal analysis, methodology, software, visualization and writing draft (DFT calculation). Z.L. assisted in funding acquisition and supervision. W.L., C.X., Q.M., and H.L. provided resources and reviewed the work. C.C. reviewed the work.

## Supporting information

Supporting Information

## Data Availability

The data that support the findings of this study are available from the corresponding author upon reasonable request.
