# Supplementary material for: High‐Density Oxygen Doping of Conductive Metal Sulfides for Better Polysulfide Trapping and Li2S‐S8 Redox Kinetics in High Areal Capacity Lithium–Sulfur Batteries
Source: Adv Sci (Weinh). 2022 Apr 11;9(17):2200840. doi: 10.1002/advs.202200840 (PMC9189686; doi:10.1002/advs.202200840)
Supplement: Supplementary file 1 — Supporting Information [file ADVS-9-2200840-s001.pdf]

ADVANCED SCIENCE
Open Access

Supporting Information

for *Adv. Sci.*, DOI 10.1002/advs.202200840

High-Density Oxygen Doping of Conductive Metal Sulfides for Better Polysulfide Trapping and Li_2S-S_8 Redox Kinetics in High Areal Capacity Lithium–Sulfur Batteries

Yiyi Li, Haiwei Wu, Donghai Wu, Hairu Wei, Yanbo Guo, Houyang Chen*, Zhijian Li*, Lei Wang, Chuanyin Xiong, Qingjun Meng, Hanbin Liu and Candace K. Chan*

Supporting Information

High-density Oxygen Doping of Conductive Metal Sulfides for Better Polysulfide Trapping and Li_2S-S_8 Redox Kinetics in High Areal Capacity Lithium-Sulfur Batteries

Yiyi Li [a1], Haiwei Wu [a1], Donghai Wu [b1], Hairu Wei [a], Yanbo Guo [a], Houyang Chen [c]*, Zhijian Li [a]*, Lei Wang [d], Chuanyin Xiong [a], Qingjun Meng [a], Hanbin Liu [a], Candace K. Chan [e]*

[a] *Shaanxi Provincial Key Laboratory of Papermaking Technology and Specialty Paper Development, College of Bioresources Chemical and Materials Engineering, Shaanxi University of Science &Technology, Xi'an, P.R. China (710021); National Demonstration Center for Experimental Light Chemistry Engineering Education, Shaanxi University of Science & Technology, Xi'an, P.R. China (710021)*

Dr. Haiwei Wu, Email: haiweiwufly@163.com, wuhaiwei@sust.edu.cn,

Prof. Zhijian Li, Email: zjli@sust.edu.cn,

[b] *Henan Key Laboratory of Nanocomposites and Applications, Institute of Nanostructured Functional Materials, Huanghe Science and Technology College, Zhengzhou 450006, China*

[c] *Chongqing Institute of Green and Intelligent Technology, Chinese Academy of Sciences, Chongqing 400714, China.*

Prof. Houyang Chen, Email: chenhouyang@cigit.ac.cn.

[d] *Shaanxi Key Laboratory of Green Preparation and Functionalization for Inorganic Materials, School of Materials Science and Engineering, Shaanxi University of Science and Technology, Xi'an 710021, China*

[e] *Materials Science and Engineering, School for Engineering of Matter, Transport and Energy, Arizona State University, Tempe, AZ (85287)*

[1] *Yiyi Li, Haiwei Wu and Donghai Wu were equal major contributors.*

Experimental Section

Materials. Chemicals were purchased from Sinopharm Chemical Reagent Co., Ltd, China. Multiwalled carbon nanotubes (MWCNT) were purchased from Beijing Deke Daojin Science and Technology Co., Ltd. Li_2S was bought from Alfa Reagent Corporation. Cellulose nanofibers (CNF) were bought from Guilin Qihong Techology Co., Ltd, China. Deionized (DI) water was produced by a Milli-Q Integral Water Purification on System in the laboratory (15 MΩ·cm).

Preparation of $NiCo_2O_4$, $NiCo_2(O\text{-}S)_4$ and $NiCo_2S_4$. Specifically, 0.6 mmol Ni $(NO_3)_2 \cdot 6H_2O$, 1.2 mmol Co $(NO_3)_2 \cdot 6H_2O$, 0.2 g nano self-made carbon spheres and 11 mmol

urea were dissolved into 80 mL of DI water and sonicated to form a homogenous solution. The solution was then transferred to a 100 mL Teflon-lined stainless-steel autoclave and kept at 180 ℃ for 10 h. After the hydrothermal reaction, the NiCo-precursors were filtered and rinsed several times with DI water. Then, the NiCo-precursors were annealed at 380 °C for 4 h with a heating rate of 1 °C min^{-1} in air, and after cooling down, the $NiCo_2O_4$ particles were collected. To perform the sulfidation of $NiCo_2O_4$ to $NiCo_2S_4$, 0.2 g $NiCo_2O_4$ (1 mmol) was immersed in 0.2 M Na_2S solution (3.9 g $Na_2S{\cdot}9H_2O$ dissolved into 80 mL of DI water) and kept at 160 °C for 8 h in a 100mL Teflon-lined stainless-steel autoclave. After cooling down naturally to room temperature, the $NiCo_2S_4$ particles were washed with deionized water, vacuum dried at 60 ℃ for 12 hours and finally collected. To prepare $NiCo_2(O\text{-}S)_4$, the reaction time and quantity of Na_2S for sulfidation of $NiCo_2O_4$ were controlled. When using 0.02 M Na_2S, the sulfidation time was varied from 1, 2, 4 and 8 h. When the sulfidation time was fixed at 8 h, the concentrations of Na_2S for sulfidation were also varied to 0.014 and 0.04 M. The optimized parameters for making $NiCo_2(O\text{-}S)_4$ were determined to be 8 h sulfidation time with 1.75 mmol Na_2S (0.02 M). The 1.75 mmol Na_2S represents a mole ratio of O (in $NiCo_2O_4$): S (in Na_2S) to be 2:1.

Preparation of Ni-OS, Ni-OP, Co-OS, Mo-OS, etc.

Preparation of MoO_3, Mo-OS and MoS_2. 4 mmol $(NH_4)_2MoO_4$ was dissolved into 20 mL of DI water and sonicated to form a homogenous solution. The pH was adjusted to 1 with 37% HCl and then the solution was transferred to a Teflon-lined stainless-steel autoclave and kept at 200 ℃ for 8 h. After the hydrothermal reaction, the precipitates were rinsed several times with DI water, dried at 80 °C overnight and finally collected as MoO_3. For preparing Mo-OS, 1.44 g MoO_3 was immersed in 1.67 M thiourea solution (1.9 g thiourea was dissolved into 15 mL of DI water) and kept at 200 °C for 18 h. After cooling down naturally to room temperature, the Mo-OS particles were washed with DI water, vacuum dried at 60 ℃ for 12 hours and finally collected. In order to prepare MoS_2, 1.44 g MoO_3 was immersed in 4.67 M thiourea solution (5.32 g thiourea was dissolved into 80 mL of DI water) and kept at 220 °C for 18 h. After cooling down naturally to room temperature, the MoS_2 particles were washed with DI water, vacuum dried at 60 ℃ for 12 hours and finally collected.

Preparation of NiO, Ni-OS and NiS. 10 mmol Ni $(NO_3)_2{\cdot}6H_2O$ was dissolved into 20 mL of

DI water to form a transparent green solution. The pH was adjusted to 12 with NaOH particles and then the solution was transferred to a Teflon-lined stainless-steel autoclave and kept at 180 ℃ for 16 h. After the hydrothermal reaction, the NiCo-precursors were filtered and rinsed several times with DI water. The Ni-precursors were first dried at 80 °C overnight and then annealed at 500 °C for 2 h with a heating rate of 3 °C min^{-1} in air, and after cooling down, the NiO particles were collected. For sulfidation of NiO to form NiS, 0.2 g NiO was immersed in 0.2 M Na_2S solution (3.9 g $Na_2S·9H_2O$ was dissolved into 80 mL of DI water) and kept at 180 °C for 8 h. After cooling down naturally to the room temperature, NiS particles were washed with deionized water, vacuum dried at 60 ℃ for 12 hours and finally collected. In order to prepare oxygen-doped NiS (Ni-OS), 0.2 g NiO was immersed in 0.02 M Na_2S solution (0.42 g $Na_2S·9H_2O$ was dissolved into 80 mL of DI water) and kept at 180 °C for 8 h. After cooling down naturally to the room temperature, the Ni-OS particles were washed with DI water, vacuum dried at 60 ℃ for 12 hours and finally collected.

Preparation of NiO, Ni-OP and Ni_2P. NiO was prepared as described above. For phosphidization of NiO to form Ni_2P, 0.2 g NiO was immersed in 0.2 M phosphorus red solution (0.5 g phosphorus red was dissolved into 80 mL of DI water) and kept at 180 °C for 10 h. After cooling down naturally to the room temperature, the Ni_2P particles were washed with deionized water, vacuum dried at 60 ℃ for 12 hours and finally collected. In order to prepare Ni-OP, 0.2 g NiO was immersed in 0.08 M phosphorus red solution (0.2 g phosphorus red was dissolved into 80 mL of DI water) and kept at 180 °C for 10 h. After cooling down naturally to the room temperature, the Ni-OP particles were washed with deionized water, vacuum dried at 60 ℃ for 12 hours and finally collected.

Preparation of Co_3O_4, Co-OS and Co_3S_4. 8 mmol Co $(NO_3)_2·6H_2O$ and 8 mmol urea were dissolved into 80 mL of DI water to form a transparent pink solution. Then, the solution was transferred to a Teflon-lined stainless-steel autoclave and kept at 200 ℃ for 24 h. After the hydrothermal reaction, the NiCo-precursors were filtered and rinsed several times with DI water. The Co-precursors were first dried at 80 °C overnight and then annealed at 500°C for 2 h with a heating rate of 3 °C min^{-1} in air, and after cooling down, the Co_3O_4 particles were collected. For sulfidation of Co_3O_4 to form Co_3S_4, 0.2 g Co_3O_4 were immersed in 0.2 M Na_2S solution (3.9 g $Na_2S·9H_2O$ was dissolved into 80 mL of DI water) and kept at 210 °C for 24 h.

After cooling down naturally to room temperature, the Co_3S_4 particles were washed with DI water, vacuum dried at 60 °C for 12 hours and finally collected. In order to prepare Co-OS, 0.2 g Co_3O_4 was immersed in 0.02 M Na_2S solution (0.42 g $Na_2S \cdot 9H_2O$ was dissolved into 80 mL of DI water) and kept at 210 °C for 24 h. After cooling down naturally to room temperature, the Co-OS particles were washed with deionized water, vacuum dried at 60 °C for 12 hours and finally collected.

Fabrication of $NiCo_2O_4$, $NiCo_2S_4$ and $NiCo_2(O\text{-}S)_4$ papers. In a typical preparation, 32.5 mg MWCNT, 5 mg CNF and 12.5 mg NiCo compounds were ultrasonically dispersed in 40 mL water and vacuum filtered to obtain a round plate with a diameter of 40 mm on the microporous membrane. After freeze-drying, free-standing $NiCo_2(O\text{-}S)_4$ (or $NiCo_2O_4$, $NiCo_2S_4$) papers were obtained with areal density of roughly 3.8 mg cm^{-2}. For preparing $NiCo_2(O\text{-}S)_4$ papers with higher areal density, 50 mg MWCNT, 7.5 mg CNF and 20 mg $NiCo_2(O\text{-}S)_4$ were used in the preparation process for areal density of roughly 7.1 mg cm^{-2}.

Preparation of the $NiCo_2(O\text{-}S)_4$ interlayer. The original $NiCo_2(O\text{-}S)_4$ was first ultrasonicated with an ultrasonic cell disrupter system. The strong ultrasonic force crushed most of the flower-like micro $NiCo_2(O\text{-}S)_4$ particles. After filtration and vacuum drying at 60 ℃, the refined $NiCo_2(O\text{-}S)_4$ was finally collected. Then, the refined $NiCo_2(O\text{-}S)_4$/carbon black/PVDF slurry was prepared by mixing 80 wt% the prepared $NiCo_2(O\text{-}S)_4$ powders, 10 wt% carbon black and 10 wt% PVDF into NMP solvent, which was then coated onto one side of a polypropylene (PP) membrane by the doctor blade casting method. The separators were then dried in air at 40 °C for 12 h to obtain a PP separator coated with $NiCo_2(O\text{-}S)_4$ interlayers. The total areal loading of the added $NiCo_2(O\text{-}S)_4$, carbon black and PVDF was around 1.0 mg cm^{-2}.

Assembly of Li-Paper/LiPS cells. For LiPS catholyte preparation, 0.56 g of sulfur and 0.115 g of Li_2S were added into 4 mL 1,3-dioxolane (DOL)/1,2-dimethoxyethane (DME) co-solvent (1:1 in volume) in a 10 mL volumetric flask. The suspension was heated at 80 °C for 48 h in an Ar-filled glove box to yield about 0.625 M Li_2S_8 (equal to 5 M elemental S) catholyte with red-brown color. For assembling Li-Paper/LiPS cells, the 40 mm papers with embedded NiCo compounds (areal density of 3.8 mg cm^{-2}) were punched into 12.5 mm discs. Various volumes

of Li_2S_8 catholyte were dropped onto the disc: 22 μL for ~3.5 mg S and areal loading equal ~2.9 mg cm^{-2}; 25 μL for ~4 mg S and areal loading ~ 3.3 mg cm^{-2}; 33 μL for ~5.25 mg S and areal loading ~ 4.4 mg cm^{-2}. Then, electrolyte (1 M LiTFSI in DME/DOL with 2% $LiNO_3$ +0.025M Li_2S_8) was added so that the total volume of Li_2S_8 catholyte and electrolyte equals to be 50 μL. The different coin cells were assembled using Li-metal as the anode. Specifically, for the cells with a sulfur loading of 2.5 mg cm^{-2}, a smaller, 10 mm diameter paper discs (areal density of 3.8 mg cm^{-2}), 12 μL Li_2S_8 catholytes and 28 μL of electrolyte were adopted to get better diffusion of LiPS on the paper discs. For the cells with a sulfur loading of 4.4 mg cm^{-2}, $NiCo_2(O\text{-}S)_4$ papers with areal density of 3.8 and 7.1 mg cm^{-2} were adopted.

The interlayer-assisted cells were prepared by using the $NiCo_2(O\text{-}S)_4$ coated PP as separator instead of PP. $NiCo_2(O\text{-}S)_4$ papers with an areal density of 7.1 mg cm^{-2} (1.6 mg cm^{-2} $NiCo_2(O\text{-}S)_4$,4.0mg cm^{-2} MWCNT and) were used. In order to make low E/S Li-S cells, the DME/DOL solvents from the Li_2S_8 catholytes were evaporated by placing the paper cathode on a 35 ℃ hotplate for 30 min in the glove box. In a typical fabrication, 55 μL Li_2S_8 (8.75 mg sulfur) or 66 μL Li_2S_8 (10.5 mg sulfur) catholyte was first dropped onto the 12.5 mm $NiCo_2(O\text{-}S)_4$ paper disc. After the DME/DOL solvents were evaporated, 40 μL of electrolyte (1 M LiTFSI in DME/DOL with 2% $LiNO_3$ +0.025M Li_2S_8), the $NiCo_2(O\text{-}S)_4$ coated PP separator, and and Li anode were assembled into the coin cell. The sulfur loading was 8.75 mg cm^{-2} (51.9 wt% in cathode + interlayer) and E/S to was 3.8 μL mg^{-1} for the cells that were prepared with 66 μL Li_2S_8 catholyte.

Materials Characterization. The microstructures of the samples were characterized with transmission electron microscopy (TEM, Tecnai F30 G2, FEI, USA) and scanning electron microscope (Hitachi S4800) equipped with an energy dispersive spectroscopy (EDS) system. Crystalline structures of the materials were characterized by X-ray diffraction using Cu kα radiation (Bruker D8 Advance, Germany) over a range of 10° < 2θ < 80° at a scan rate of 4° min^{-1}. The electronic conductivity was measured using the four-probe method and carried out using RTS-9 (4 PROBES TECH, Guangzhou, China). Before testing, the powder samples were pressed into cylindrical sheets ($NiCo_2O_4$, NCo_2S_4 and $NiCo_2(O\text{-}S)_4$ sheets) under 25 MPa. X-ray photoelectron spectroscopy (XPS) measurements were carried out on an ESCALAB 250 spectrometer with Mg and Ka radiation. For the Li_2S_6 adsorption test, a 5 mM

(or 10mM) Li_2S_6 solution was prepared by mixing S and Li_2S with a molar ratio of 1:5 in a DME and DOL mixture (1:1 v/v). About 15 mg of the as-prepared catalyst compounds were added into 10 mL of the Li_2S_6 solution, then rested for 6 h.

Electrochemical Characterization. Electrochemical tests were obtained by using standard CR2032 coin-type half-cells. A multi-channel galvanostatic system (LAND CT2000A) was used to evaluate the electrochemical performance. Electrochemical charge and discharge tests were conducted from 1.7 to 2.8 V (vs Li^+/Li). The cyclic voltammetry (CV) and electrochemical impedance spectroscopy (EIS) measurements were measured with an electrochemical workstation (PARSTAT 4000+). The CV was measured over the range of 1.7-2.8 V (vs Li^+/Li) with a step of 0.1 mV s^{-1} while EIS was tested with an amplitude of 10 mV and a frequency range of 0.01 Hz to 100 kHz. Symmetric cells were assembled with $NiCo_2O_4$, $NiCo_2S_4$ and $NiCo_2(O\text{-}S)_4$ papers as the electrodes and 5 mol L^{-1} Li_2S_6 in DME/DOL (1:1 in volume) as the electrolyte. The amount of electrolyte added into each cell was 50 µL. CV curves of the symmetric cells were performed at scan rates of 1, 5 and 10 mV s^{-1} over the range of -1.5 to 1.5 V, respectively. Li_2S nucleation tests were carried out by using 5 M Li_2S_8 as catholyte, $NiCo_2O_4$, $NiCo_2S_4$ and $NiCo_2(O\text{-}S)_4$ papers as current collectors, and lithium foil as anode. For the test, 30 µL of Li_2S_8 catholyte was added to the papers. These cells were first discharged at a current of 0.1 mA until the voltage reached 2.10 V vs. Li/Li^+; then, the voltage was held at 2.07 V until the current decreased to 10^{-2} mA.

Theoretical details. To further investigate the interplay between lithium s ulfides and O-doped $NiCo_2S_4$, spin-polarized density functional theory (DFT) calculations were performed using the Vienna Ab-initio Simulation Package (VASP),[1] with the projector augmented wave method (PAW).[2] The exchange-correlation potential was described by the generalized gradient approximation of Perdew-Burke-Ernzerhof (GGA-PBE)[3] with a cutoff energy of 450 eV for the expansion of electronic wave function in the plane waves basis set. The atomic positions were fully relaxed until the total energy and Hellmann-Feynman forces were less than 10^{-5} eV and 0.02 eV/Å, respectively. The Brillouin-zone integrations were performed with a Gaussian smearing of 0.1 eV over a 1 × 2 × 1 Monkhorst-Pack k-point mesh for the surface slab model.

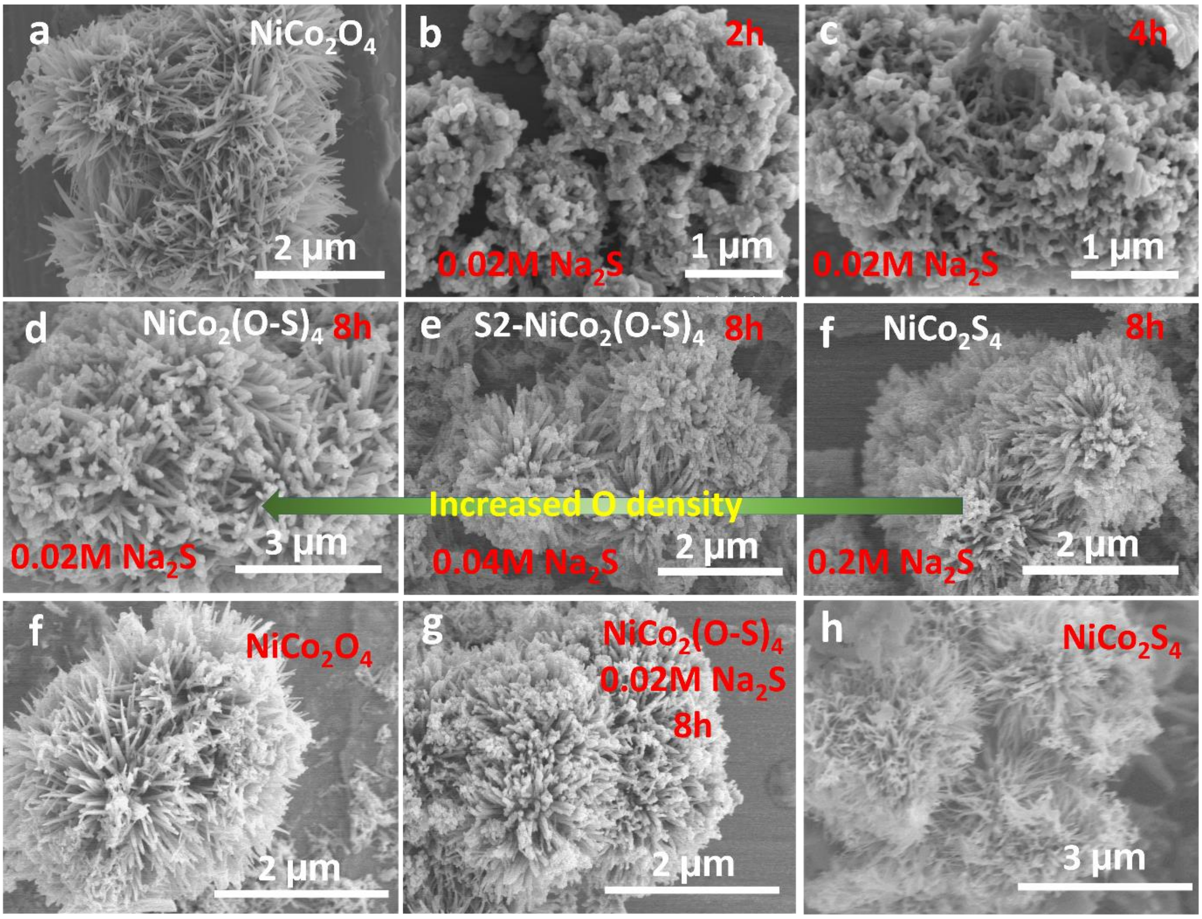

Figure S1 SEM images of pristine $NiCo_2O_4$ (a), $NiCo_2(O\text{-}S)_4$ powders obtained after sulfidation of $NiCo_2O_4$ with 0.02 M Na_2S for 2 h (b), 4 h (c) and 8 h (d). SEM image of flower-like S2-$NiCo_2(O\text{-}S)_4$ after further sulfidation with 0.04 M Na_2S (e). SEM image of $NiCo_2S_4$ after sulfidation with 0.2 M Na_2S (f). Additional SEM images of $NiCo_2O_4$ (f), $NiCo_2(O\text{-}S)_4$ (g) and $NiCo_2S_4$ (h).

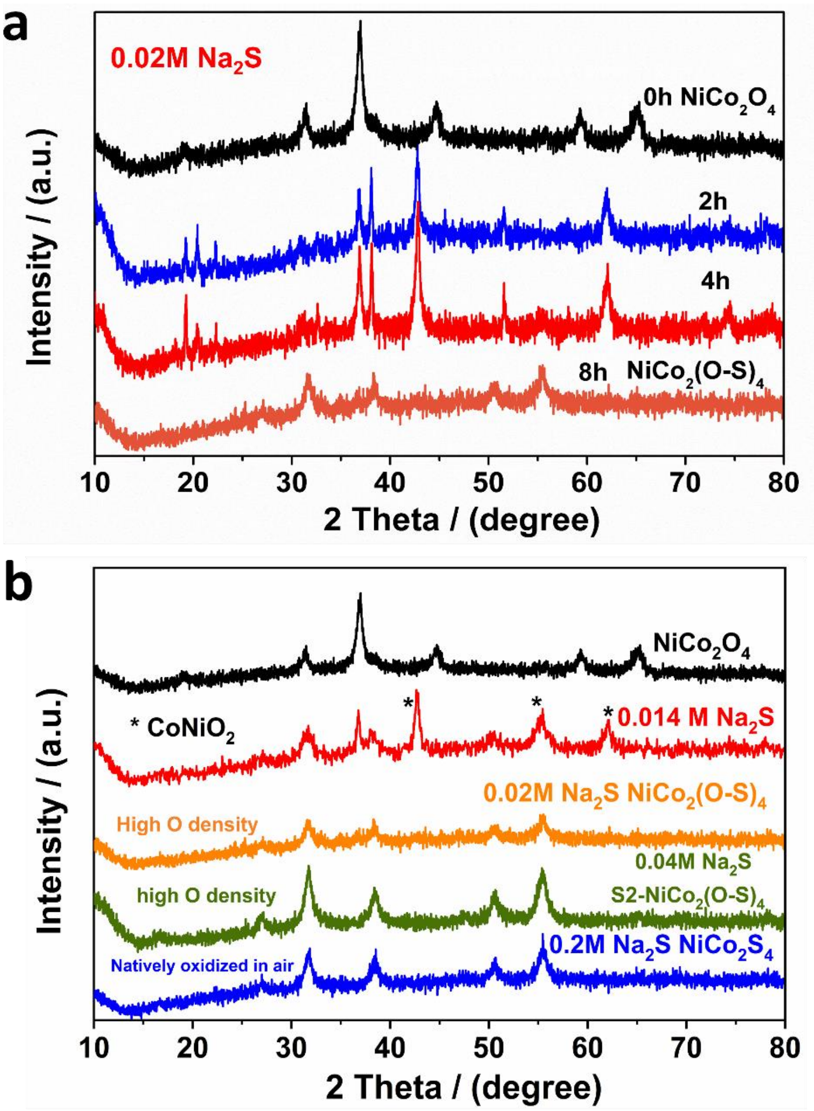

Figure S2 XRD patterns of $NiCo_2O_4$ samples after sulfidation with 0.02 M Na_2S but different reaction times (a). XRD patterns of $NiCo_2O_4$ samples with sufficient sulfidation time (8h) but different amounts of Na_2S (different mole concentration with same volume of 80 mL) (b). 0.02 M Na_2S corresponding to mole ratio of $NiCo_2O_4$: Na_2S =1:2 and targeted theoretical atom ratio of O:S in O dopped $NiCo_2S_4$ can be 1:1; 0.04M Na_2S corresponding to mole ratio of $NiCo_2O_4$: Na_2S =1:4 and targeted theoretical atom ratio of O:S in O dopped $NiCo_2S_4$ can be 1:0.5; 0.2M Na_2S corresponding to mole ratio of $NiCo_2O_4$: Na_2S =1:20 and $NiCo_2O_4$ can be fully sulfurized to be $NiCo_2S_4$.$NiCo_2(O\text{-}S)_4$ and S2-$NiCo_2(O\text{-}S)_4$ have higher O content than pristine $NiCo_2S_4$, which is natively oxidized in air.

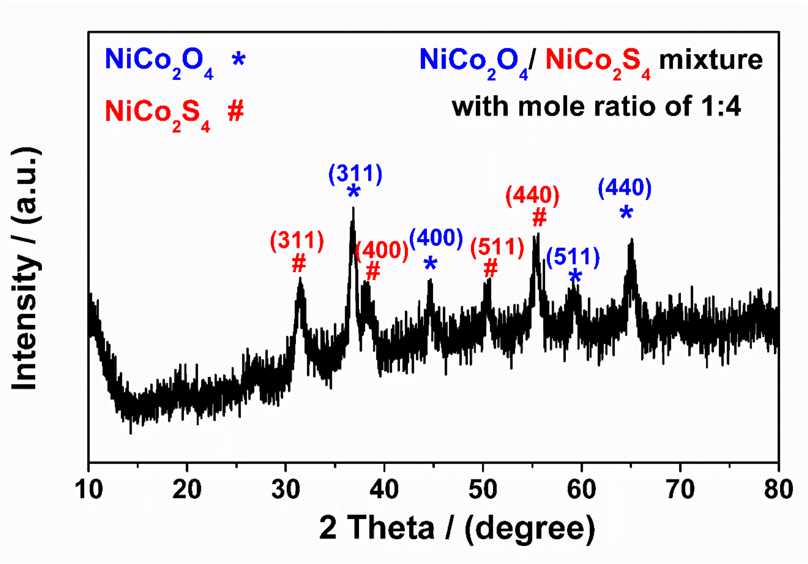

Figure S3 XRD pattern of $NiCo_2O_4$/$NiCo_2S_4$ mixture

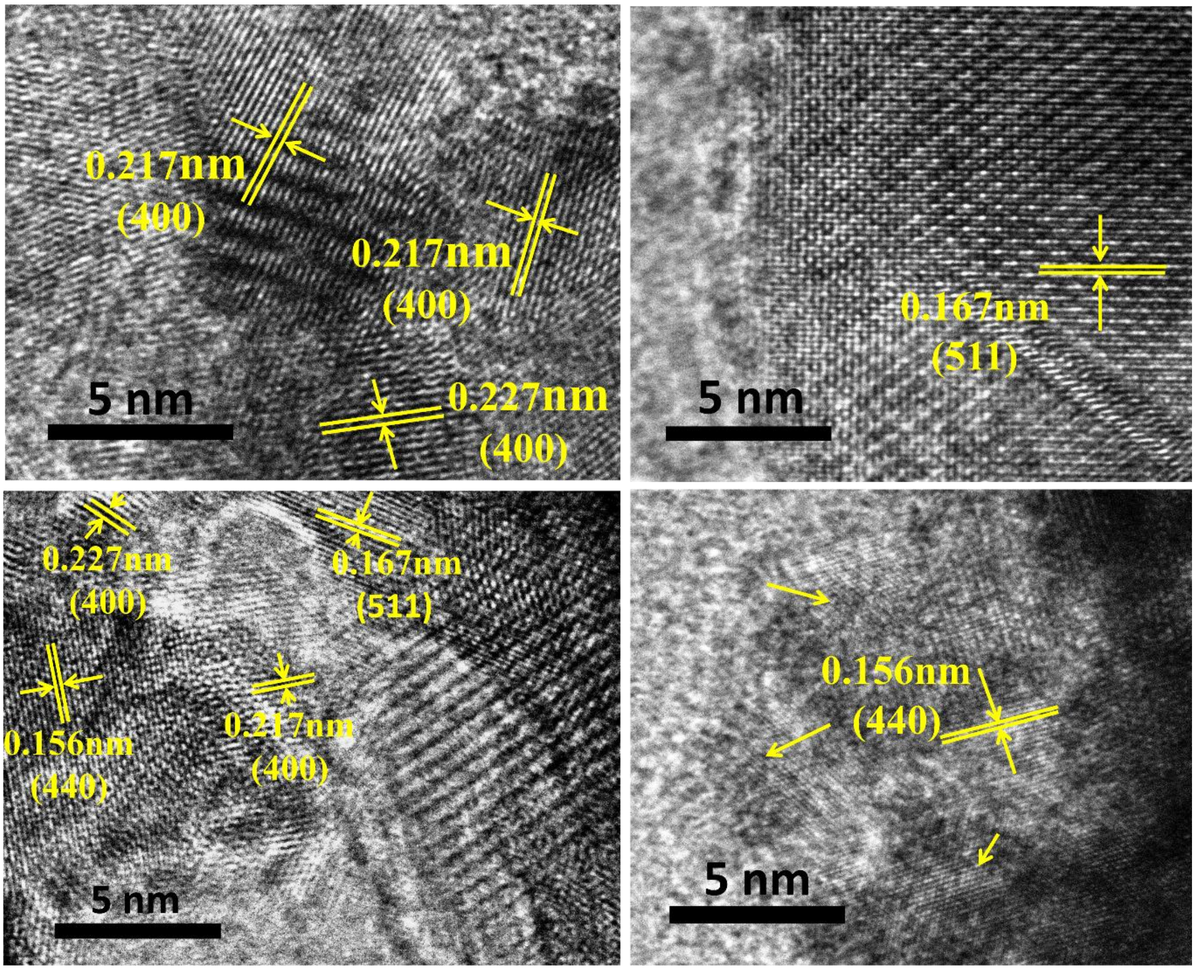

Figure S4 HRTEM images of $NiCo_2(O\text{-}S)_4$

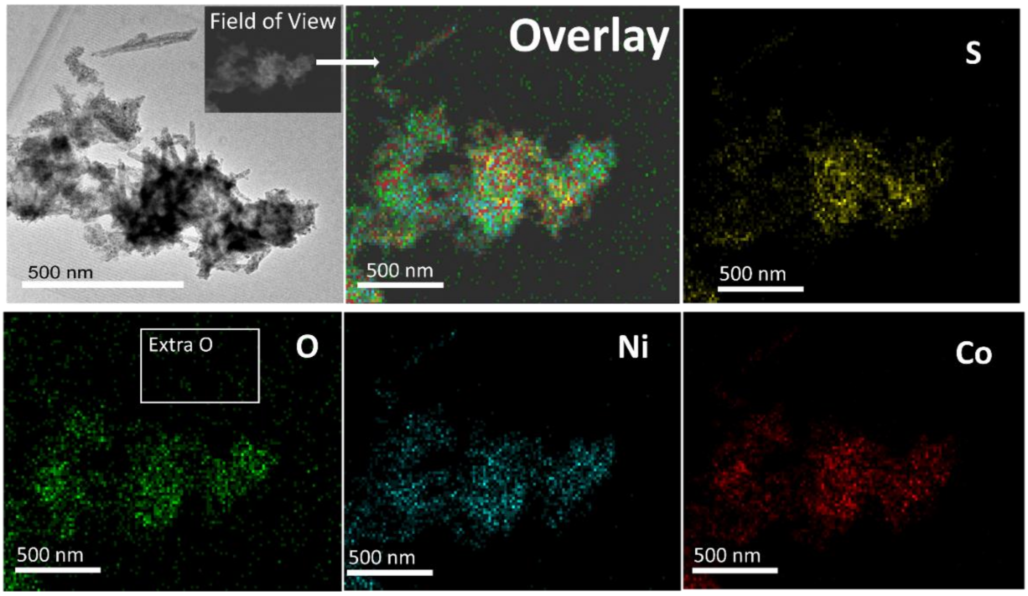

Figure S5 EELS elemental mappings of $NiCo_2(O\text{-}S)_4$

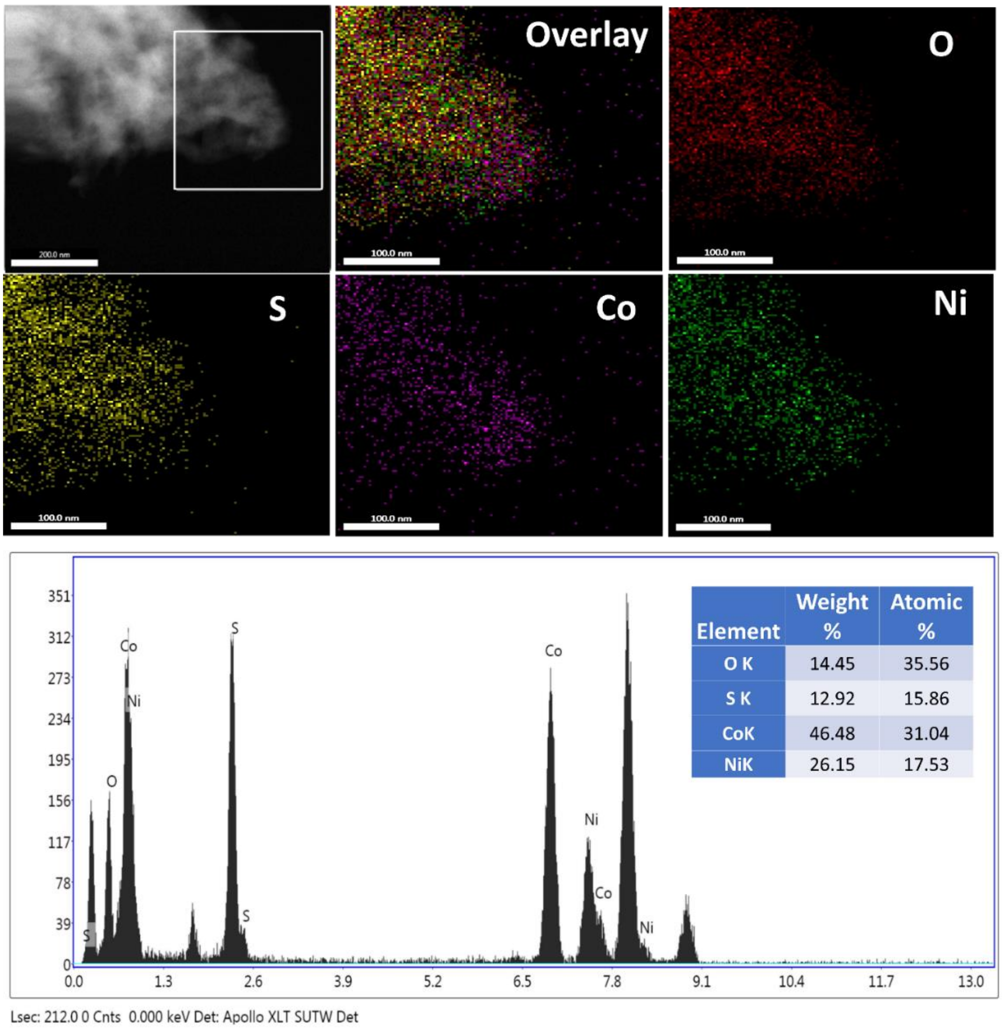

Figure S6 Additional EELS elemental mappings and spectrum of $NiCo_2(O\text{-}S)_4$, verifying a high-density O-doped surface.

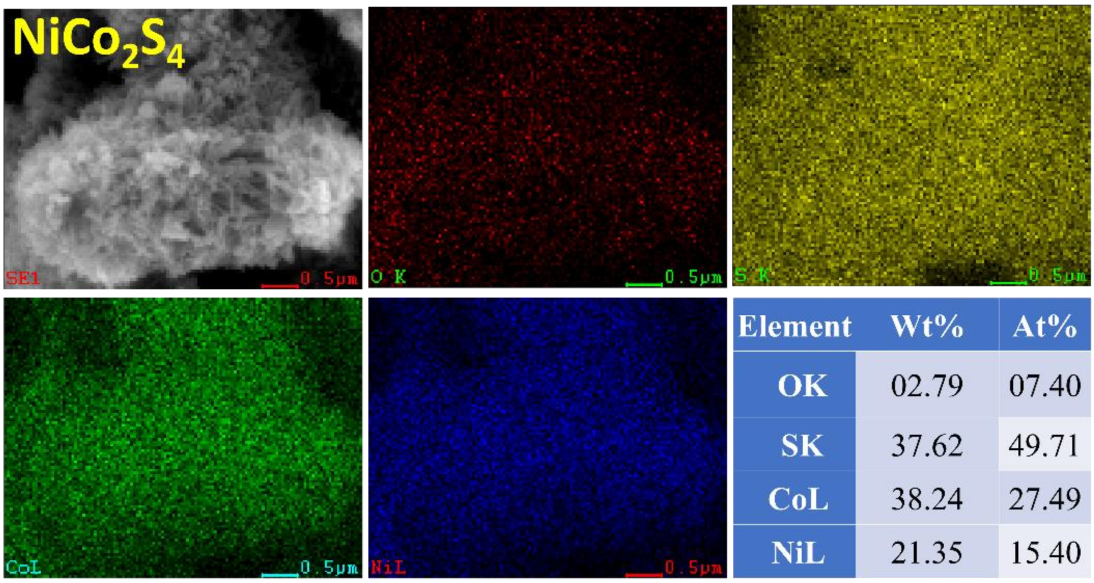

Element	Wt%	At%
OK	02.79	07.40
SK	37.62	49.71
CoL	38.24	27.49
NiL	21.35	15.40

Figure S7 EDS elemental mappings of $NiCo_2S_4$, showing $NiCo_2S_4$ was oxidized mildly in air and exhibiting a O-S co-existed surface which is similar with mildly oxidized CoP in Wang's work [1].

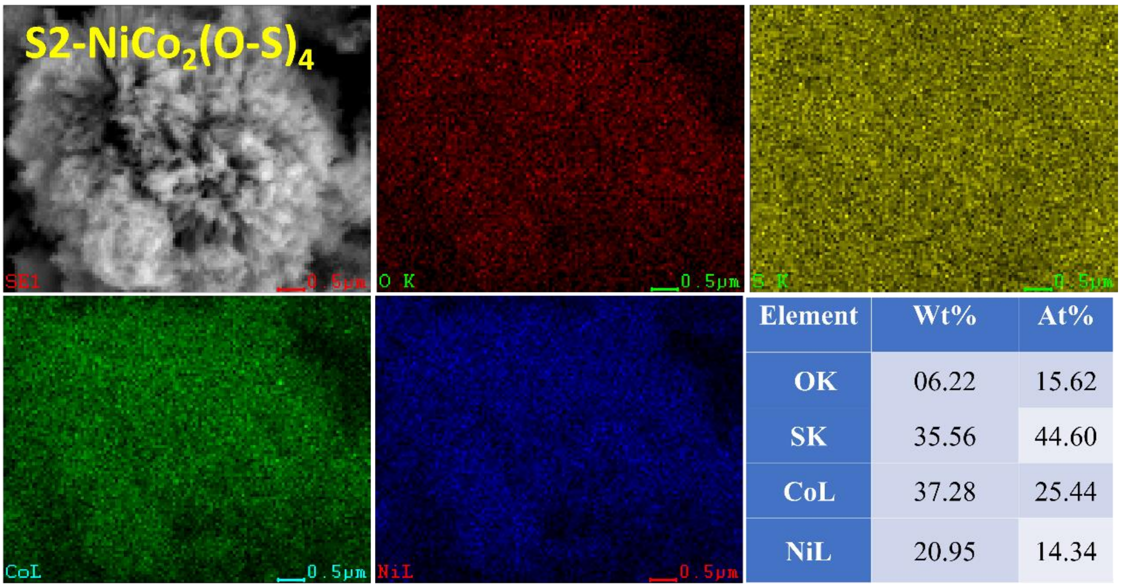

Element	Wt%	At%
OK	06.22	15.62
SK	35.56	44.60
CoL	37.28	25.44
NiL	20.95	14.34

Figure S8 EDS elemental mappings of S2-$NiCo_2(O\text{-}S)_4$, showing a little bit higher-density oxygen dopped surface than $NiCo_2S_4$ in Figure S7.

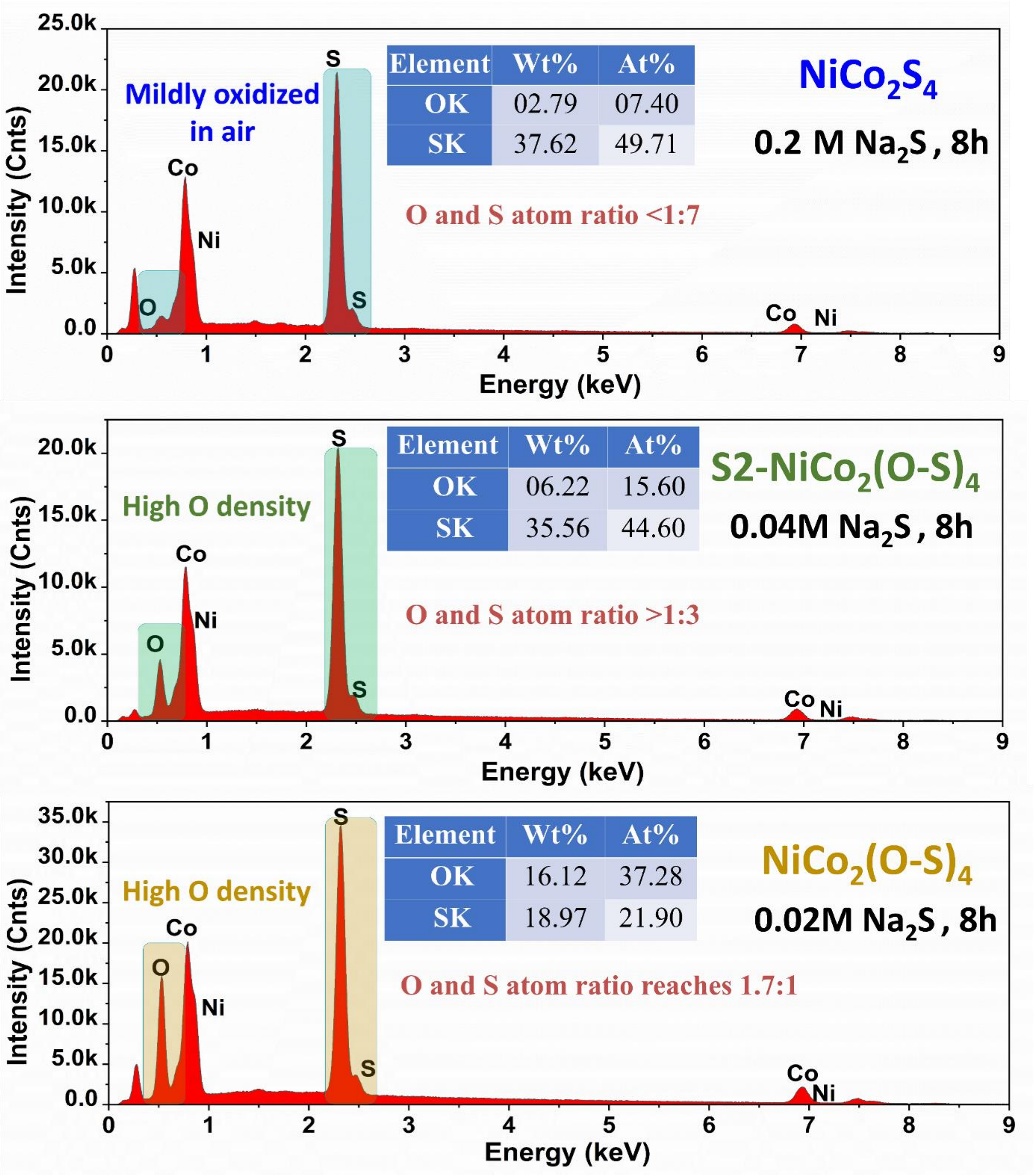

Figure S9 EDS spectra of $NiCo_2S_4$, S2-$NiCo_2(O\text{-}S)_4$ and $NiCo_2S_4$, showing an increased amount of oxygen content.

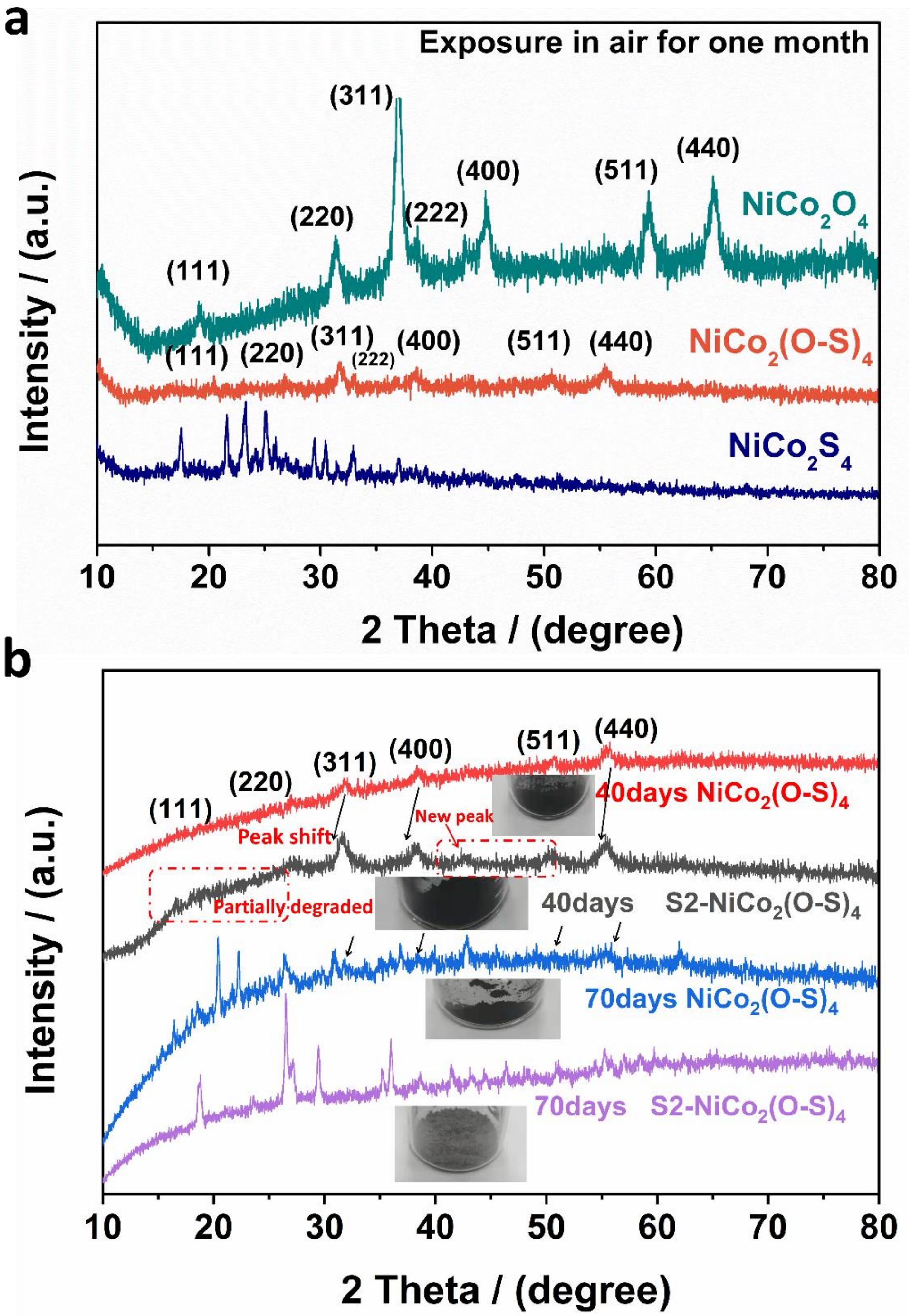

Figure S10 XRD patterns of $NiCo_2O_4$, $NiCo_2(O\text{-}S)_4$ and $NiCo_2S_4$ after exposure in air for one month (a). XRD patterns and optical images of $NiCo_2(O\text{-}S)_4$ and S2-$NiCo_2(O\text{-}S)_4$ after exposure in air for 40 days and 70days (b). Noting that after 40 days, the typical peaks of S S2-$NiCo_2S_4$ show left shift compared to $NiCo_2(O\text{-}S)_4$ and some new peaks start to appear. After 70 days, S2-$NiCo_2(O\text{-}S)_4$ powers turns to grey pink and show strong new peaks, indicating that it is totally degraded. $NiCo_2(O\text{-}S)_4$ still shows week peeks of original $NiCo_2(O\text{-}S)_4$ phase and keeps black, suggesting it is not totally degraded yet.

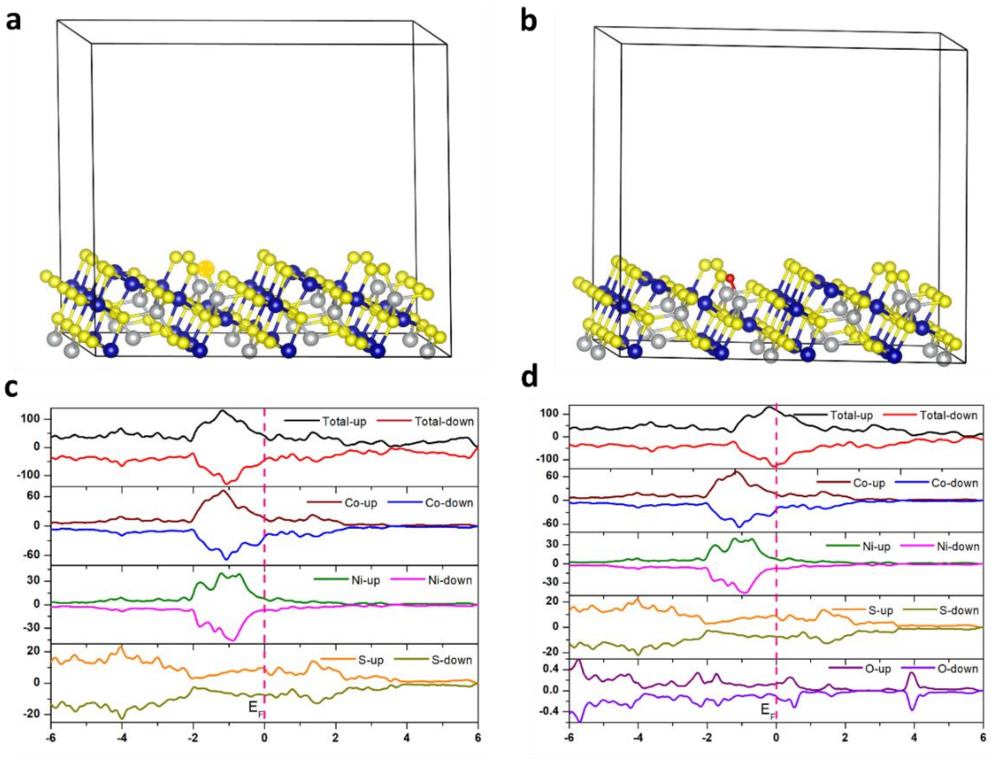

Figure S11 The periodic slab models used for pristine (a) and O-doped (b) $NiCo_2S_4$(311) surfaces. The density of states (DOS) of pristine (c) and O-doped (d) $NiCo_2S_4$ surfaces, in which the Fermi levels were set to zero with vertical dash lines.

A slab model with four layers was adopted to describe the $NiCo_2S_4$ (311) surface (Figure S11a). The upper half layers of Co/Ni and S atoms in the supercell were relaxed, while the lower half were fixed initially as those in the bulk with optimized lattice parameters of 9.263 Å (the experimental results is 9.387 Å). The supercell consists of 16 $NiCo_2S_4$ units (i.e. 112 atoms in total), and periodically repeated slabs are separated by a vacuum space of 15 Å. By comparing the total energies, we screen out the energetically most favorable configuration of O-doped $NiCo_2S_4$ by substituting the outermost (projecting most outwards) S with one O atom (O-$NiCo_2S_4$), as shown in Figure S11b. One can see that, after doping, the $NiCo_2S_4$ (311) surface almost keeps its initial morphological integrity.

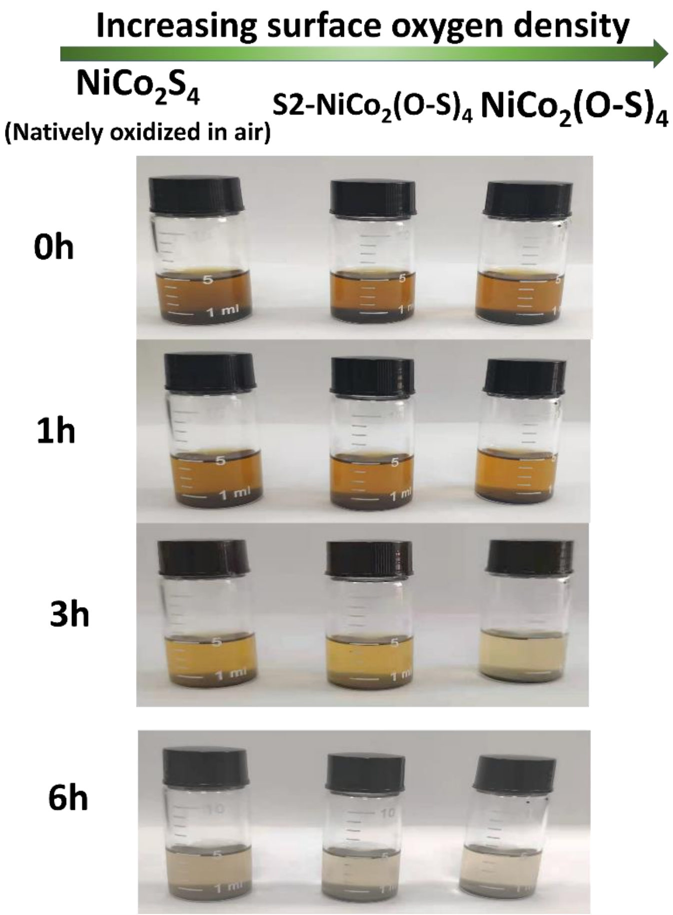

Figure S12 Optical images of $NiCo_2S_4$, S2-$NiCo_2$(O-S)$_4$ and $NiCo_2$(O-S)$_4$ soaked in the 10 mM Li_2S_6 solution

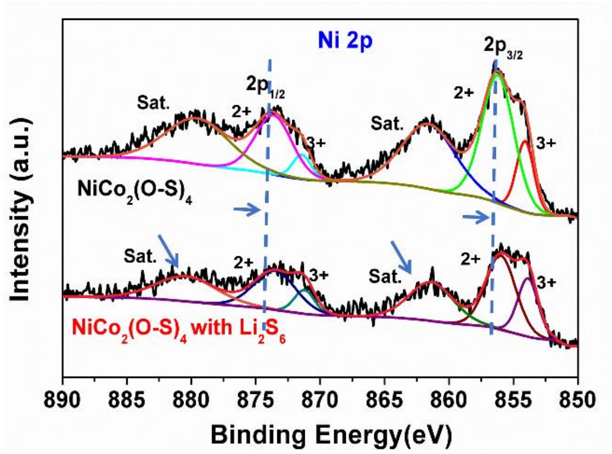

Figure S13 High-resolution XPS spectra of Ni 2p for $NiCo_2$(O-S)$_4$ with and without Li_2S_6 catholyte adsorption

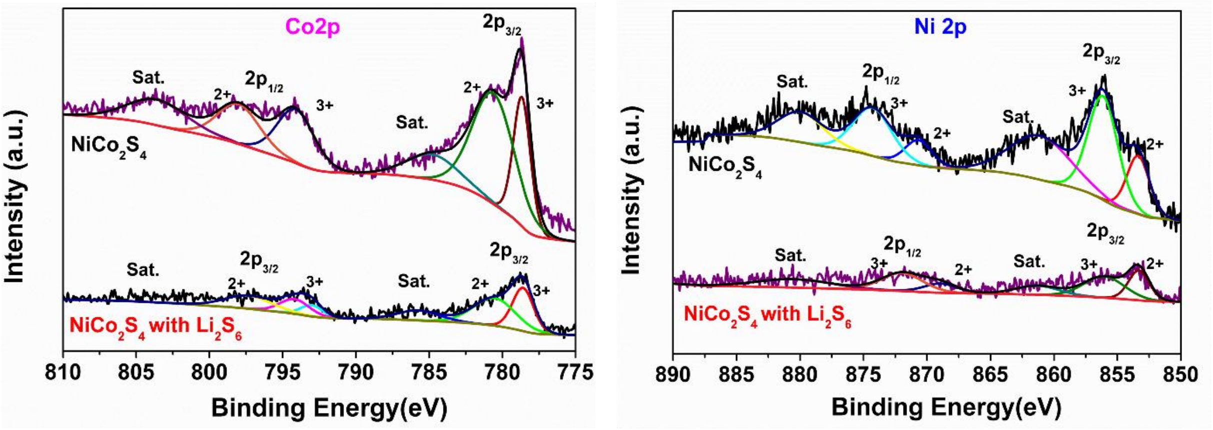

Figure S14 High-resolution XPS spectra of Co 2p and Ni 2p for $NiCo_2S_4$ with and without Li_2S_6 catholyte adsorption

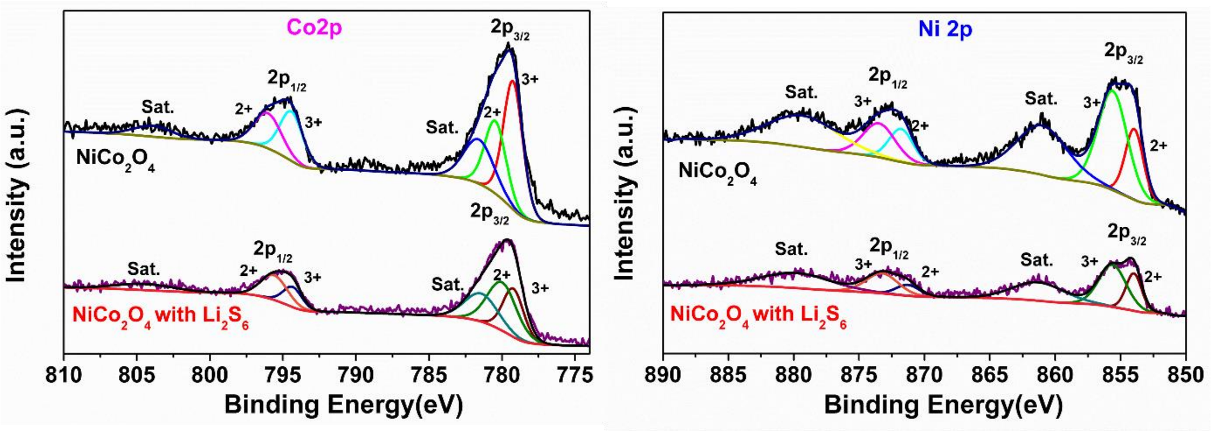

Figure S15 High-resolution XPS spectra of Co 2p and Ni 2p for $NiCo_2O_4$ with and without Li_2S_6 catholyte adsorption

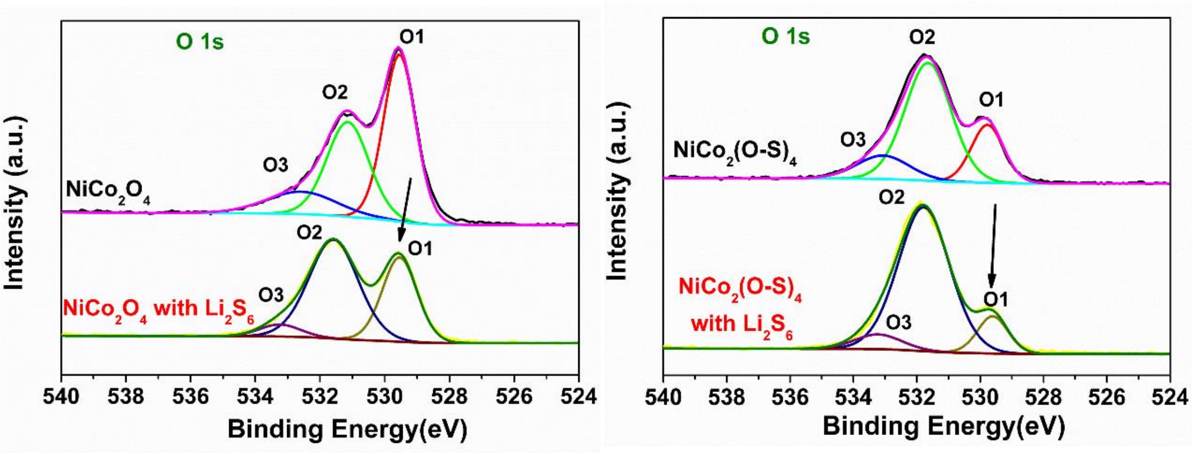

Figure S16 High-resolution XPS spectra of O 1s for $NiCo_2O_4$ and $NiCo_2(O\text{-}S)_4$ with and without Li_2S_6 catholyte adsorption

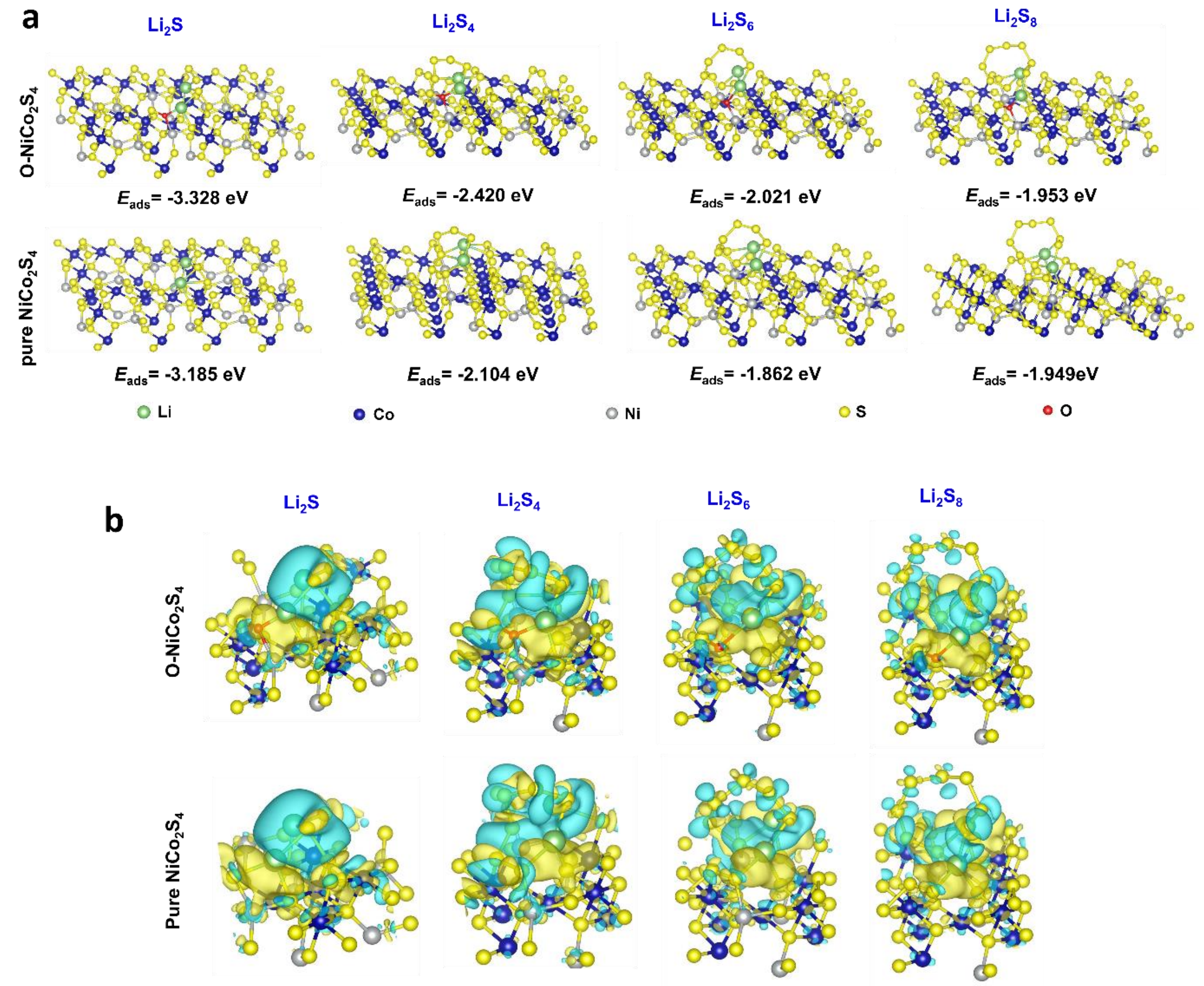

Figure S17 Optimized geometries of the Li_2S_6 and Li_2S molecule adsorbed on the $NiCo_2(O\text{-}S)_4$ (311) and $NiCo_2S_4$ surfaces, with corresponding binding energy values listed below the models (a). The charge density difference for the most stable adsorption configurations of Li_2S, Li_2S_4, Li_2S_6, Li_2S_8 on a pristine $NiCo_2S_4$ (311) surface. The charge density difference for the most stable adsorption configurations of Li_2S, Li_2S_4, Li_2S_6, Li_2S_8 on an $NiCo_2(O\text{-}S)_4$ (311) surface (b). The yellow and cyan regions represent charge accumulation and depletion, respectively. An isosurface of 0.001 e/Å^3 is set.

The calculated E_{ads} are used to find the most stable adsorption configurations for Li_2S, Li_2S_4, Li_2S_6, and Li_2S_8 on $NiCo_2(O\text{-}S)_4$ (311) and $NiCo_2S_4$ (311) surfaces (Figure S17). The results show that these lithium sulfides prefer to adsorb on the $NiCo_2S_4$ (311) surfaces perpendicularly with two Li binding with surrounding S atoms, locating above the groove constituting by two columns of S atoms. The adsorption energies for the lithium sulfides on

the O-$NiCo_2S_4$ (311) surface are more negative than those for the pristine $NiCo_2S_4$ (311) surface, implying enhanced adsorption of lithium sulfides on the O-$NiCo_2S_4$ (311) surface. The charge density difference is also plotted to better understand the bonding feature. As shown in Figure S17, the charges are mainly aggregated around the S and O atoms, while the Li atoms lost their electrons, indicating the charges are transferred from Li to S/O atoms. From the charge accumulation position around S/O and Li atoms in Figure S17, one can speculate the S-S bonds exhibit covalent character in Li_2S_n and the Li-S/Li-O bonds show some ionic bond character. The charge accumulation profiles almost enclose the O atoms; correspondingly, only small areas of charge depletion appear at the corresponding S atoms on the pristine $NiCo_2S_4$(311) surface.

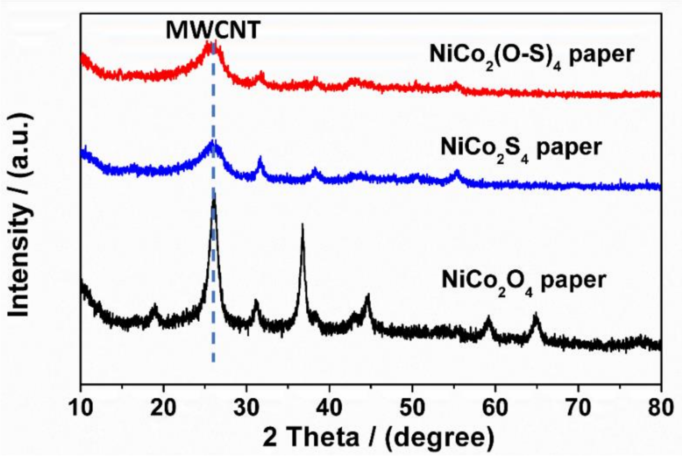

Figure S18 XRD patterns of $NiCo_2O_4$, $NiCo_2(O\text{-}S)_4$ and $NiCo_2S_4$ papers prepared with MWCNTs and cellulose nanofibers.

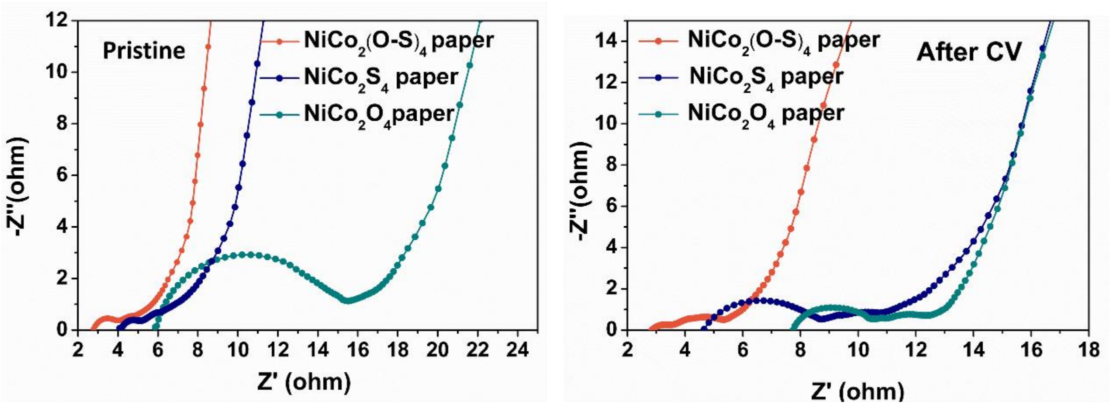

Figure S19 EIS patterns of symmetric cells with $NiCo_2O_4$, $NiCo_2(O\text{-}S)_4$ and $NiCo_2S_4$ papers before and after CV tests

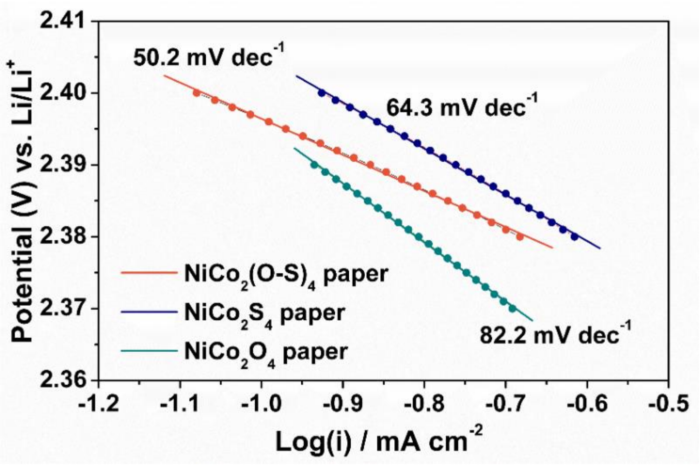

Figure S20 Tafel plots calculated from the CV curves for the high plateau reduction peak at around 2.30 V

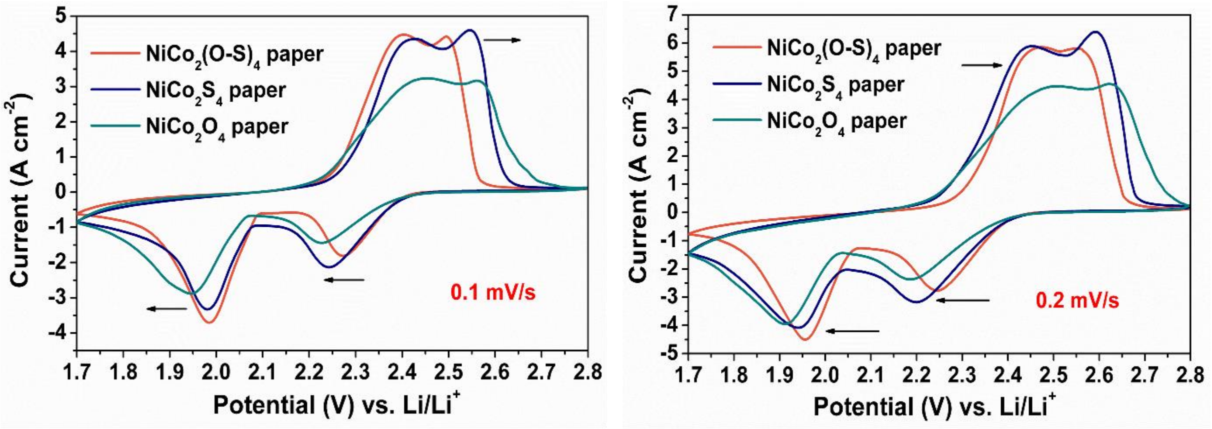

Figure S21 Comparison of CV curves at 0.1 and 0.2 mV/s for $NiCo_2O_4$, $NiCo_2(O\text{-}S)_4$ and $NiCo_2S_4$ paper supported cells

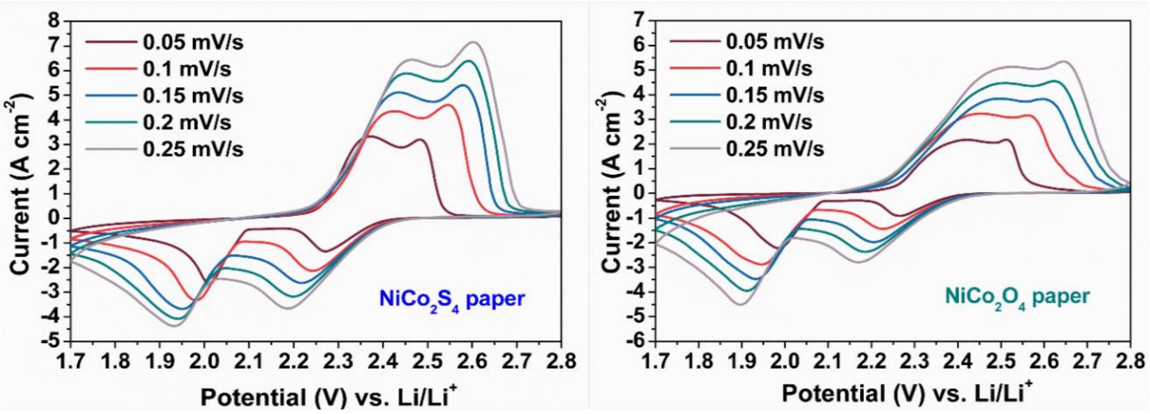

Figure S22 CV curves of $NiCo_2S_4$ and $NiCo_2O_4$ paper supported cells from 0.05 to 0.25 mV/s

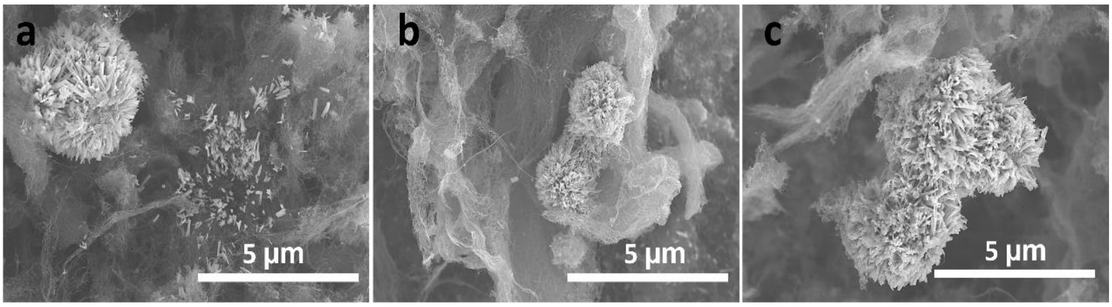

Figure S23 SEM images of pristine $NiCo_2O_4$, $NiCo_2(O\text{-}S)_4$ and $NiCo_2S_4$ papers

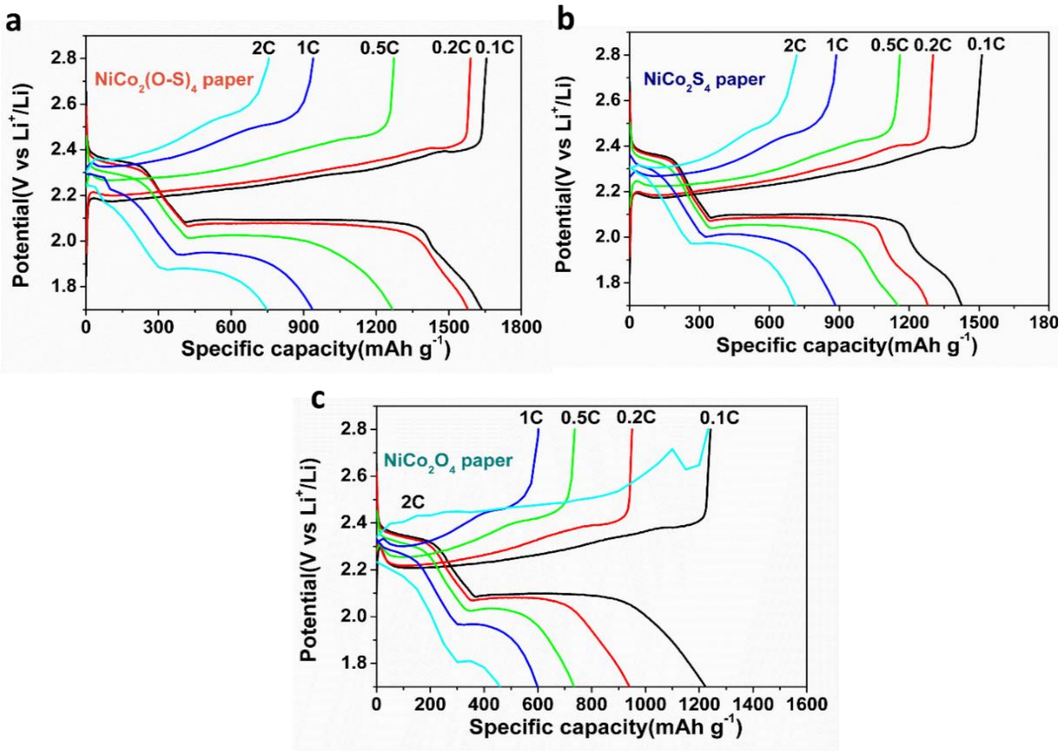

Figure S24 Discharge-charge curves of the $NiCo_2(O\text{-}S)_4$ (a), $NiCo_2S_4$ (b) and $NiCo_2O_4$ (c) paper supported cells with sulfur loading of 2.5 mg cm^{-2} (40.0 wt.% in cathode) from 0.1C to 2C.

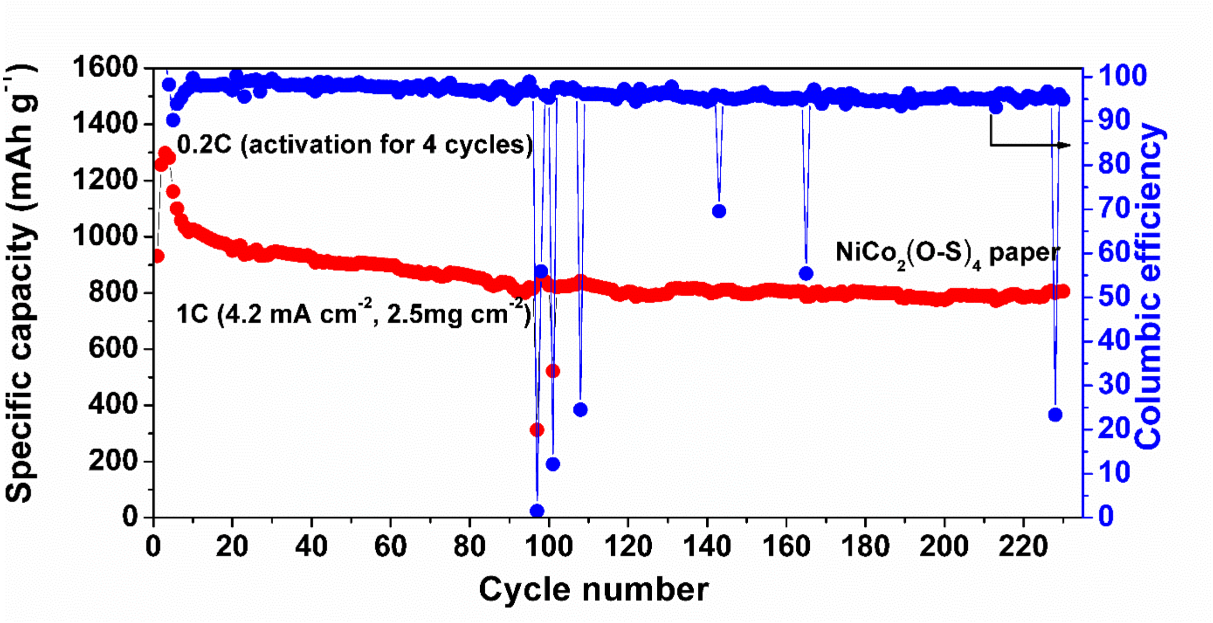

Figure S25 Cycling performance of $NiCo_2(O\text{-}S)_4$ paper supported cell with sulfur loading of 2.5 mg cm^{-2} (40.0 wt.% in cathode) at 1C.

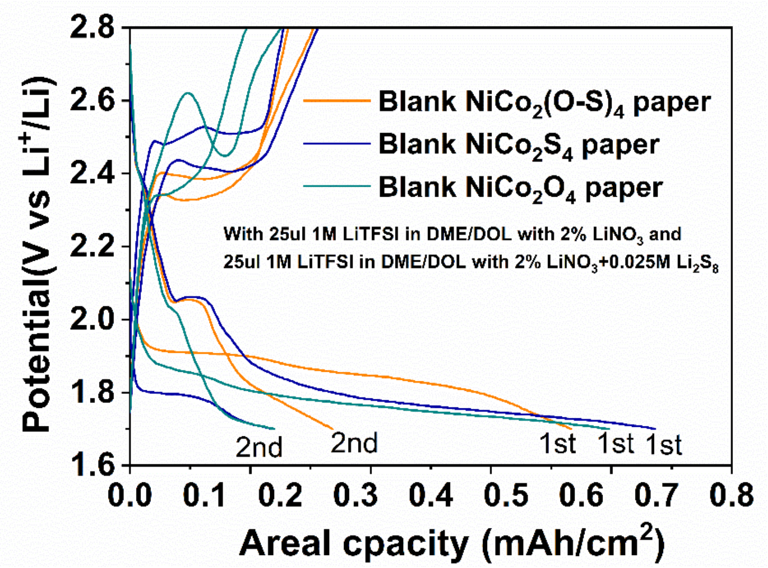

Figure S26 Discharge-charge curves of the blank $NiCo_2(O\text{-}S)_4$, $NiCo_2S_4$ and $NiCo_2S_4$ papers with 25uL 1M LiTFSI in DME/DOL with 2% $LiNO_3$ and 25uL 1M LiTFSI in DME/DOL with 2% $LiNO_3$+0.025M Li_2S_8 at 0.16 mA cm^{-2}

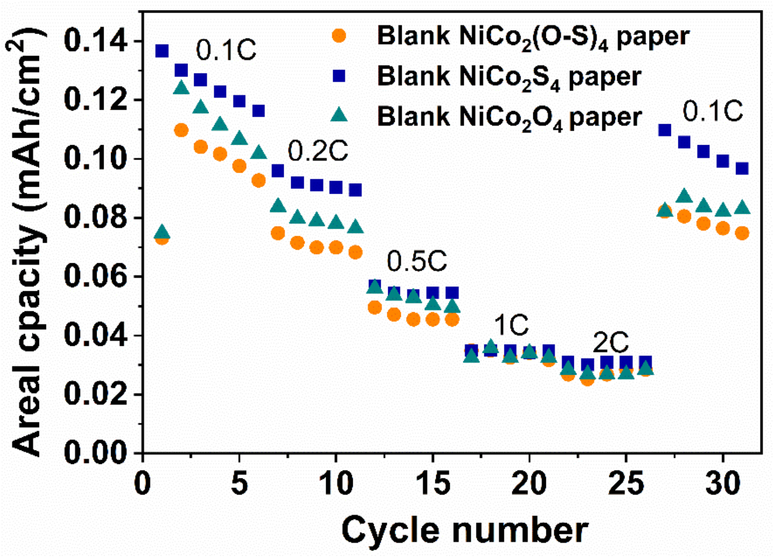

Figure S27 Rate capacities of blank $NiCo_2(O\text{-}S)_4$, $NiCo_2S_4$ and $NiCo_2S_4$ papers with 25uL 1M LiTFSI in DME/DOL with 2% $LiNO_3$ and 25uL 1M LiTFSI in DME/DOL with 2% $LiNO_3$+0.025M Li_2S_8 after activation

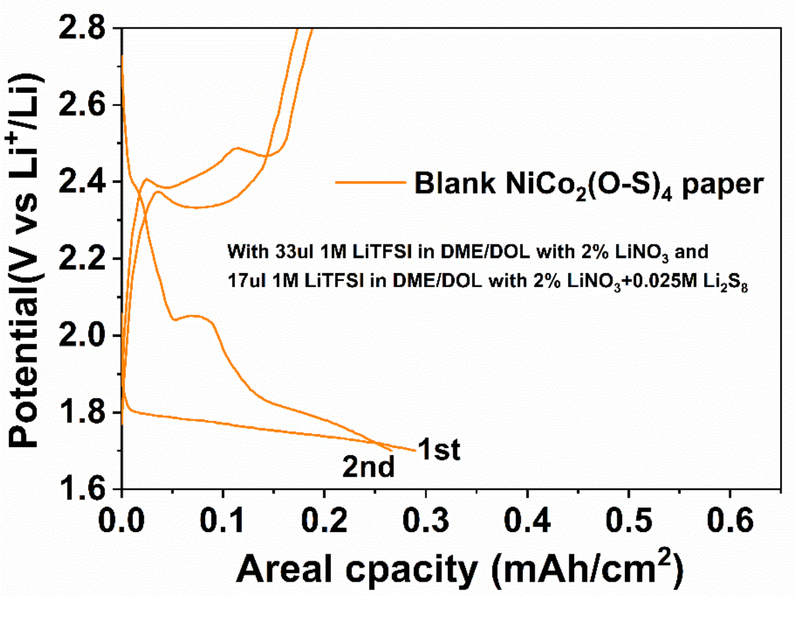

Figure 28 Discharge-charge curves of the blank $NiCo_2(O\text{-}S)_4$ paper with 33uL 1M LiTFSI in DME/DOL with 2% $LiNO_3$ and 17uL 1M LiTFSI in DME/DOL with 2% $LiNO_3$+0.025M Li_2S_8 at 0.16 mA cm^{-2}

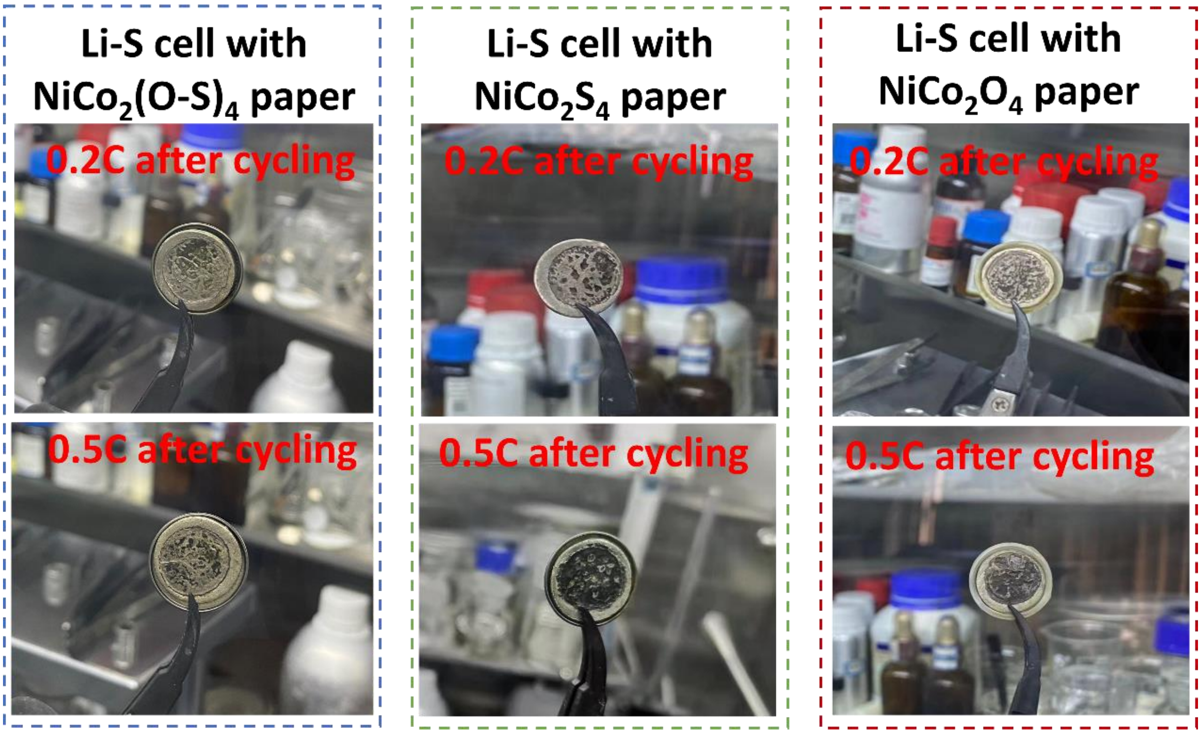

Figure S29 Li anodes from Li-S cells with sulfur loading of 2.9 mg cm^{-2} after cycling at 0.2C and 0.5C.

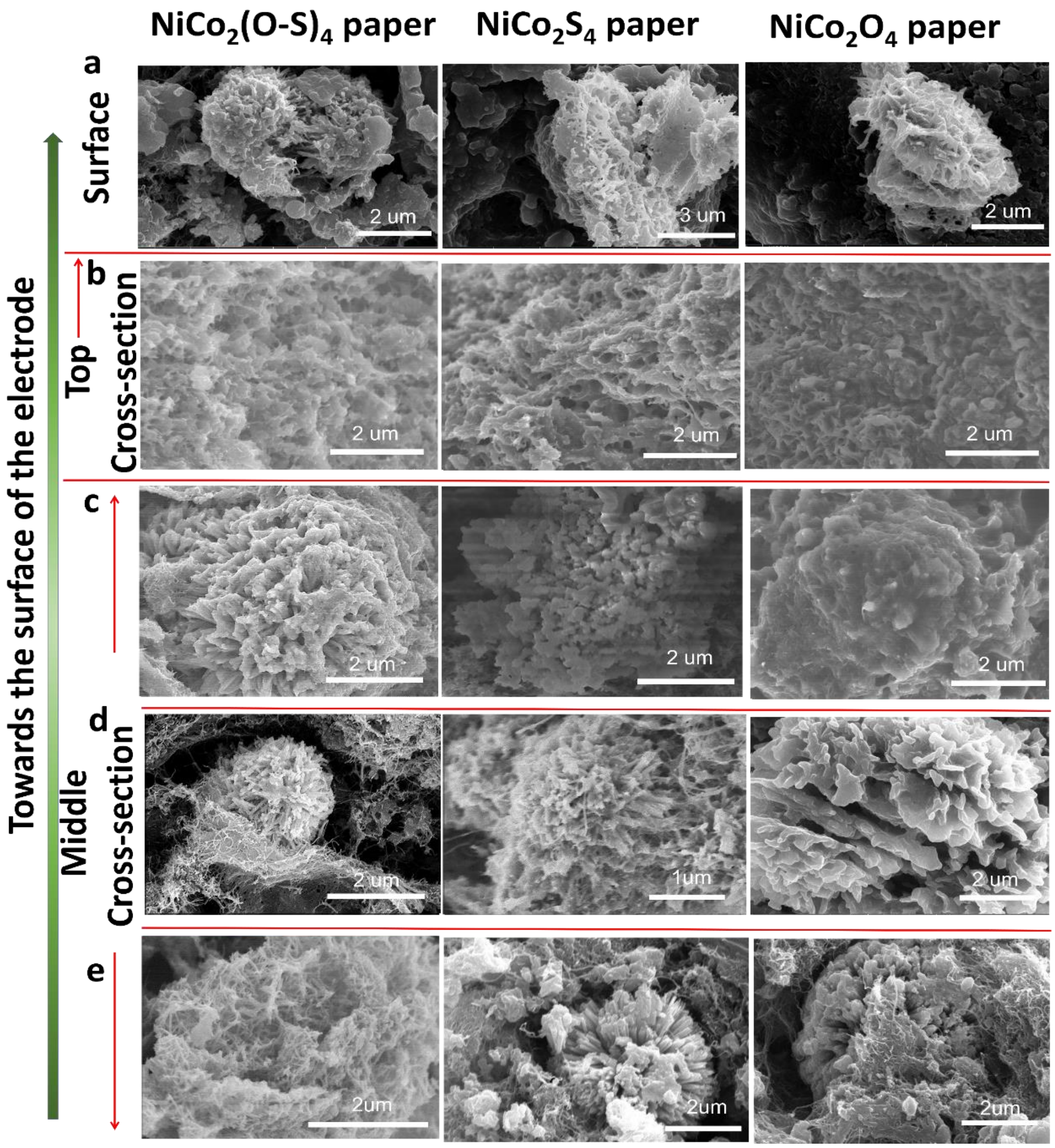

Figure S30 SEM images of electrodes cycled at 0.2C in Li-S cells with sulfur loading of 2.9 mg cm^{-2}.

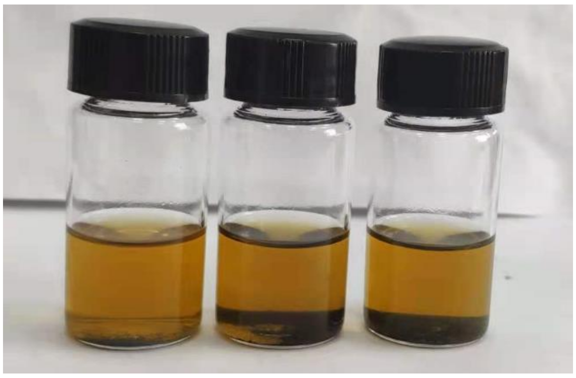

Figure S31 Optical images of 5 mL of 5 mM Li_2S_6 solution at 0 h.

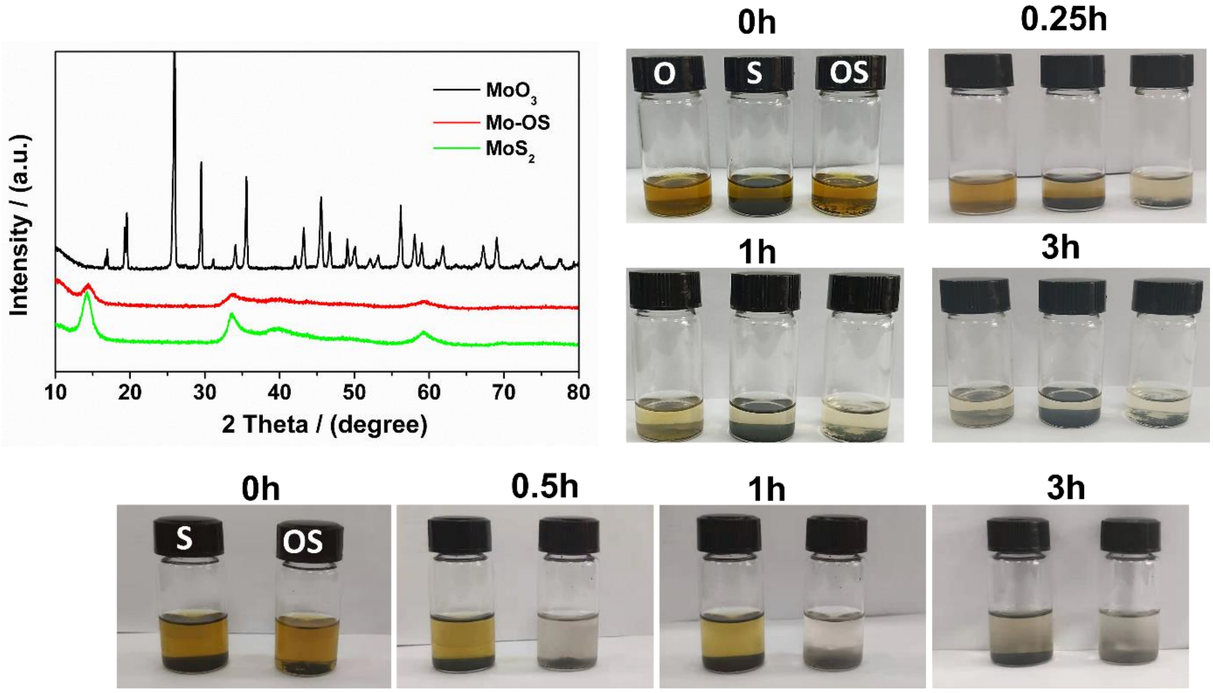

Figure S32 XRD pattern and optical images (15 mg samples to be soaked in the 5 mL of 5 mM Li_2S_6 solution) of MoO_3, Mo-OS and MoS_2.Noting that MoS_2 is natively oxidized in air while Mo-OS has a higher O content as shown in Figure S33.

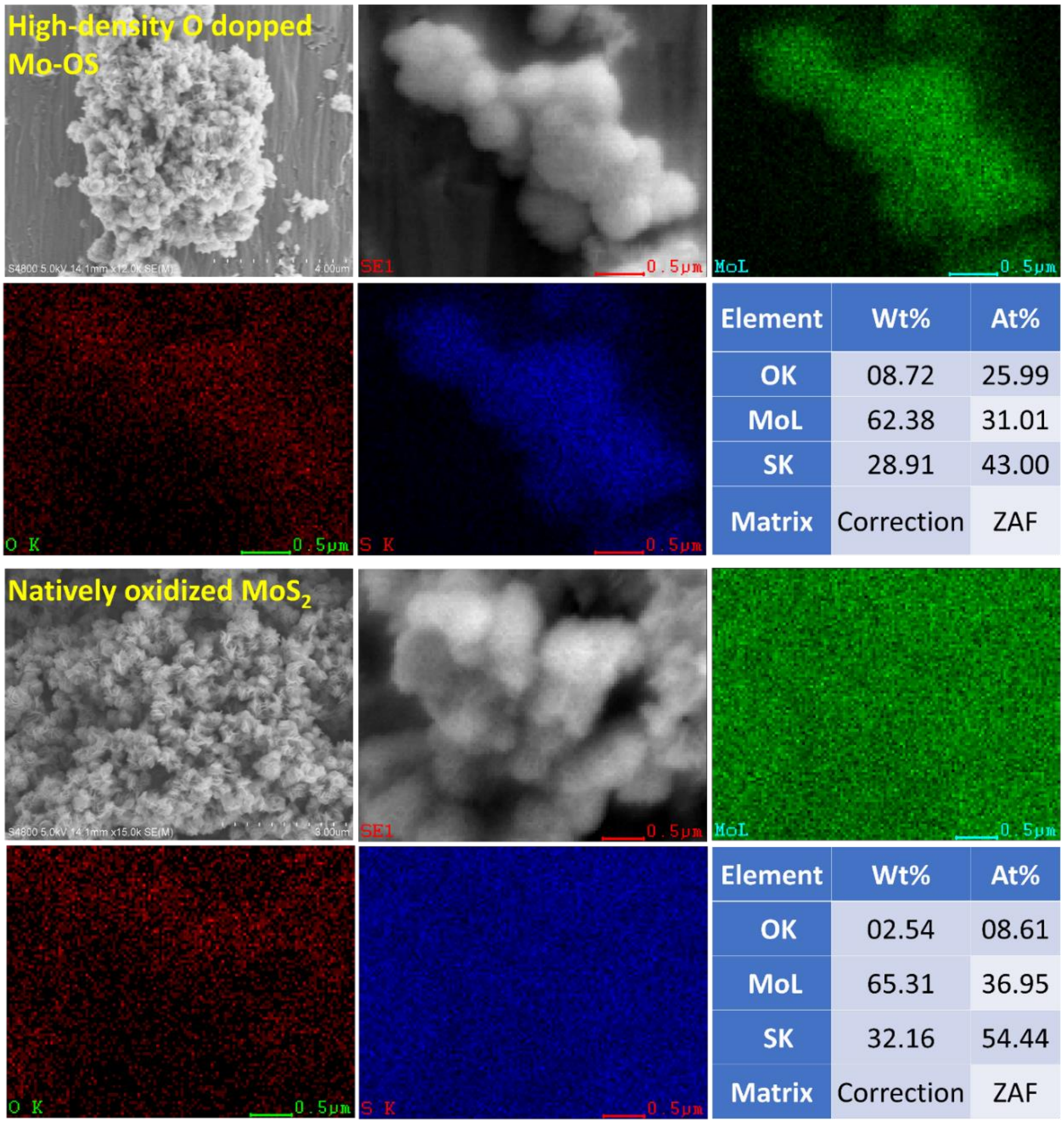

Element	Wt%	At%
OK	08.72	25.99
MoL	62.38	31.01
SK	28.91	43.00
Matrix	Correction	ZAF

Element	Wt%	At%
OK	02.54	08.61
MoL	65.31	36.95
SK	32.16	54.44
Matrix	Correction	ZAF

Figure S33 SEM and EDS images of Mo-OS and MoS_2.

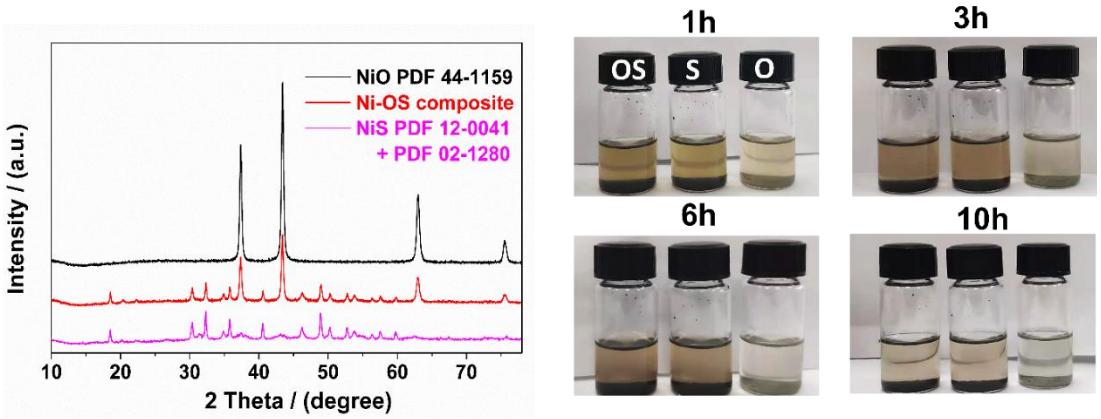

Figure S34 XRD pattern and optical images (15 mg samples to be soaked in the 5 mL of 5 mM Li_2S_6 solution) of NiO, Ni-OS and NiS. Noting that NiS is natively oxidized in air while Ni-OS has a higher O content as shown in Figure S35.

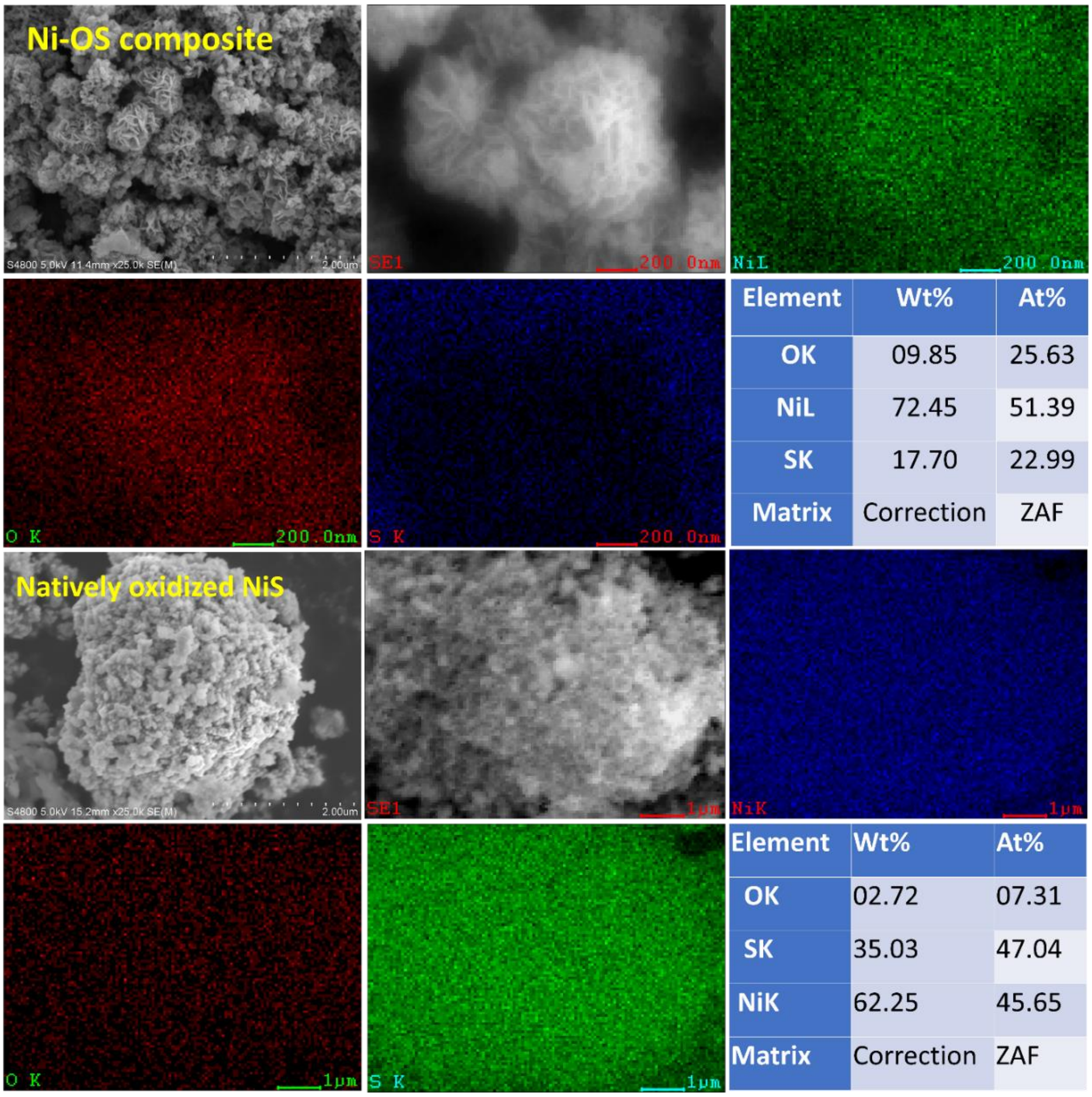

Figure S35 SEM and EDS images of Ni-OS and NiS.

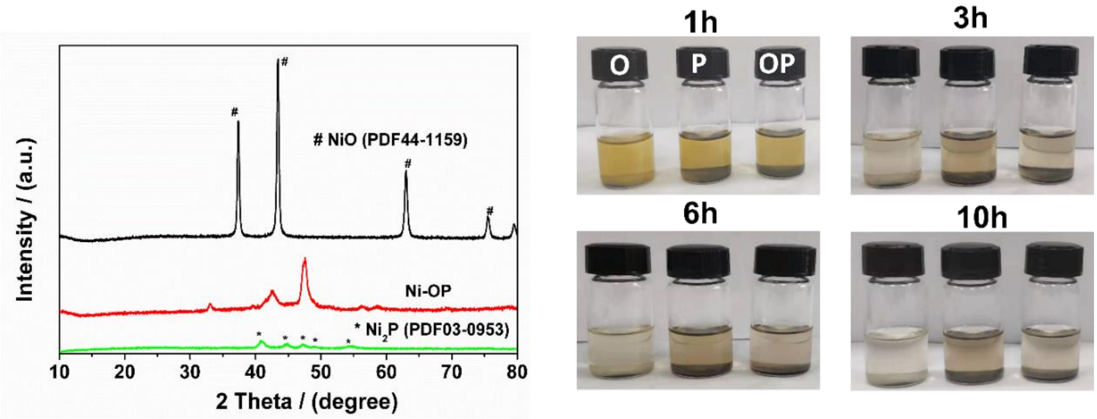

Figure S36 XRD pattern, optical images (15 mg samples to be soaked in the 5 mL of 5 mM Li_2S_6 solution) of NiO, Ni-OP and Ni_2P. Noting that Ni_2P is natively oxidized in air while Ni-OP has a higher O content as shown in Figure S37.

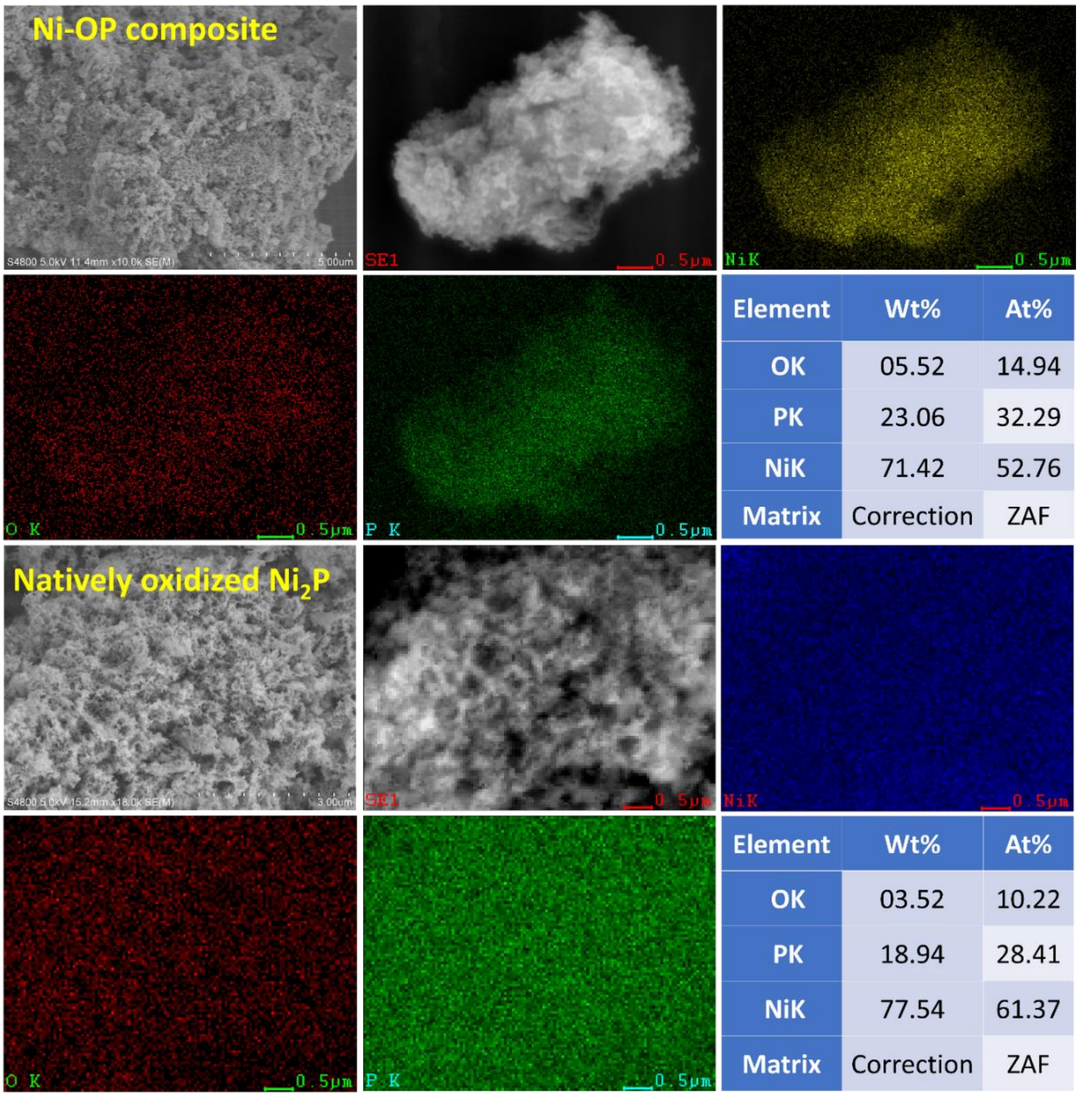

Element	Wt%	At%
OK	05.52	14.94
PK	23.06	32.29
NiK	71.42	52.76
Matrix	Correction	ZAF

Element	Wt%	At%
OK	03.52	10.22
PK	18.94	28.41
NiK	77.54	61.37
Matrix	Correction	ZAF

Figure S37 SEM and EDS images of Ni-OP and Ni_2P.

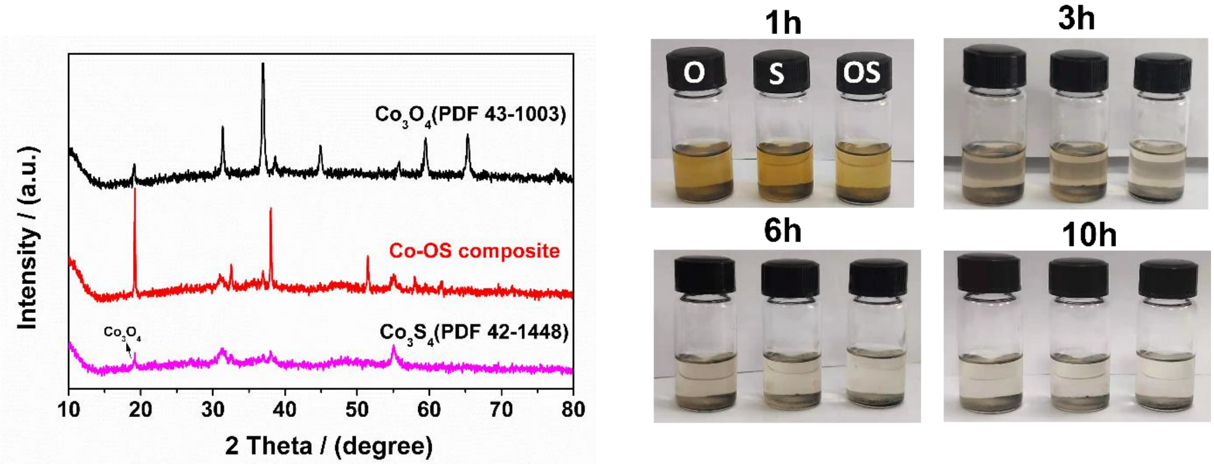

Figure S38 XRD pattern, optical images (15 mg samples to be soaked in the 5 mL of 5 mM Li_2S_6 solution) of Co_3O_4, Co-OS and Co_3S_4. Noting that Co_3S_4 is natively oxidized in air while Co-OS has a higher O content as shown in Figure S39.

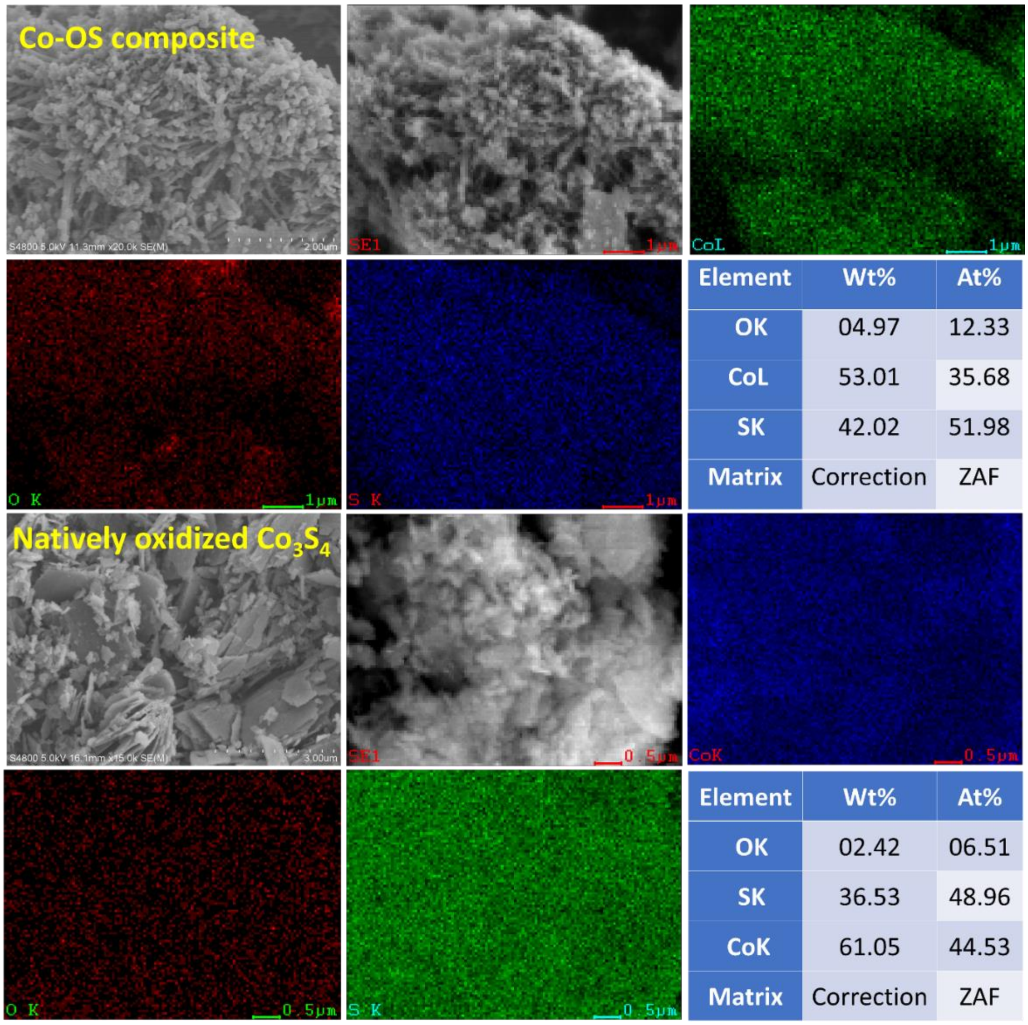

Element	Wt%	At%
OK	04.97	12.33
CoL	53.01	35.68
SK	42.02	51.98
Matrix	Correction	ZAF

Element	Wt%	At%
OK	02.42	06.51
SK	36.53	48.96
CoK	61.05	44.53
Matrix	Correction	ZAF

Figure S39 SEM and EDS images of Co-OS and Co_3S_4.

Table S1 Lattice spacings of different samples ($NiCo_2O_4$, $NiCo_2(O\text{-}S)_4$ and $NiCo_2S_4$).

Phase	$NiCo_2O_4$ Lattice spacings (nm)	$NiCo_2(O\text{-}S)_4$ Lattice spacings (nm)	$NiCo_2S_4$ Lattice spacings (nm)
**(111)**	0.469	-	0.542
**(220)**	0.287	-	0.332
**(311)**	0.245	0.313	0.283
**(400)**	0.203	0.227(0.217)	0.235
**(511)**	0.156	0.167	0.181
**(440)**	0.143	0.156	0.166

Table S2 Bader charge analysis data for $NiCo_2S_4$ (311) and O-$NiCo_2S_4$ (311)

Polysulfides	Total transferred electrons to $NiCo_2S_4$ (311) (|e|)	Total transferred electrons to O-$NiCo_2S_4$ (311) (|e|)	Gained electrons in S atom for $NiCo_2S_4$ (311) (|e|)	Gained electrons in O atom for O-$NiCo_2S_4$ (311) (|e|)
**Li_2S**	1.167	1.17	1.202	0.392
**Li_2S_4**	0.909	0.938	1.171	0372
**Li_2S_6**	0.593	0.582	1.127	0.304
**Li_2S_8**	0.677	0.727	1.133	0.309

Table S3 Comparison of the electrochemical performance of our work and other works with $NiCo_2S_4$ or $NiCo_2O_4$ as sulfur hosts

Sulfur hosts (Including interlayers)	Sulfur fraction in cathode (wt.%)	E/S ratio (ul/mg)	Sulfur utilization (%)	Sulfur loading (mg cm^{-2})	Areal capacity after cycling (mAh cm^{-2})	Current density (C and mA cm^{-2})	Cycling number	Ref
**$NiCo_2S_4$/N-CNT**	N/A	N/A	56.2%	2.1	1.97	0.2C/N/A	100	2
	N/A	N/A	43.6%	3.8	2.78	0.2C/N/A	100	
	N/A	N/A	34.3%	5.2	2.98	0.2C/N/A	100	
**MWCNT/S+$NiCo_2S_4$@CT interlayer**	8.6%	20.0	65.8%	1.5	1.65	0.1C/0.25	100	3
	15.3%	20.0	41.8%	3.1	About 2.2	0.5C/2.6	100	
	20.2%	20.0	28.7%	5.2	2.5	0.5C/4.3	100	
**CNT/S+ $NiCo_2S_4$ film interlayer**	64.3%	10.0	35.8%	2.0	About 1.2	0.5C/1.7	200	4
	65.6%	10.0	31.7%	3.4	About 1.8	0.5C/2.8	200	
	66.3%	10.0	29.6%	5.3	2.63	0.5C/4.4	200	
**CNT/S+ $NiCo_2S_4$/EOG /CNF layer**	39.1%	16.0	62.8%	2.0	About 2.1	0.1-0.3C<br>/0.3-1.0	100	5
	56.2%	11.0	49.3%	4.0	About 3.3	0.1-0.3C/<br>0.67-2.0	100	
	65.8%	9.8	45.0%	6.0	4.5	0.1-0.3C<br>1-3	100	
**CNT-S/CC @$NiCo_2O_4$**	<<0.56	N/A	39.5%	3.5	2.3	0.2C/1.2	200	6
	<<0.56	N/A	42.9%	3.76	About 2.7	0.2C/1.3	70	
	<<0.56	N/A	48.0%	6.22	About 5.0	0.2C/2.1	70	
	<0.56	N/A	47.0%	8.90	About 7.0	0.2C/3.0	70	
**S/$NiCo_2O_4$ nanofiber**	52.5%	10	54.1%	1.5	About 1.4	0.5C/1.3	100	7
	**52.5%**	**5**	**54.1%**	**1.5**	**About 1.4**	**0.1C/0.25**	**100**	
	52.5%	25	39.9%	4.0	About 2.7	0.1C/0.67	100	
**Hollow $NiCo_2O_4$/S**	53%	N/A	54.8%	N/A	N/A	0.2C/ N/A	100	8
**S/$NiCo_2O_4$ nanoflower**	33.6%	N/A	36.5%	1.15-<br>1.5	N/A	0.5C/ N/A	100	9
**Free-standing $NiCo_2(O\text{-}S)_4$**	**43.2%**	**14.3**	**57.8%**	**2.9**	**2.80**	**0.2C/1.0**	**200**	**This work**
	**43.2%**	**14.3**	**68.5%**	**2.9**	**3.32**	**0.2C/1.0**	**100**	
	**43.2%**	**14.3**	**55.5%**	**2.9**	**2.69**	**0.5C/2.4**	**150**	
	**46.5%**	**12.5**	**73.0%**	**3.3**	**3.565**	**0.15C/0.8**	**100**	
	**40.0 %**	**9.52**	**58.5%**	**4.4**	**4.28**	**0.11C/0.8**	**100**	

Table S4 Comparison of the electrochemical performance of Li-S batteries with different catalysts in this work and other recent papers.

**Catalysts**	**Sulfur content in cathode (wt.%)**	**E/S ratio (ul/mg)**	**Sulfur utilization (%)**	**Sulfur loading (mg cm^{-2})**	**Areal capacity after cycling (mAh cm^{-2})**	**Current density (C and mA cm^{-2})**	**Cycling number**	**Ref**
**MoS_2-MoN**	57.1%	12	49.2%	6.4	5.26	0.2C/2.14	100	10
	72.7%	8.3	51.2%	12.2	10.3	0.1C/10.2	100	
**3DOM Co_3O_4/CoP**	52.5%	8.8	62.7%	3.4	3.56	0.1C/0.57	100	
	52.5%	5.6	50.0%	5.3	4.43	0.1C/0.89	100	11
	52.5%	4.7	41.1%	6.4	4.40	0.1C/1.07	100	12
**MoS_2-C**	21.7%	3.7	61.1%	4.4	4.5	0.5C/3.67	160	
**Fe/Co-N**	70.0%	15.0	64.4%	6.5	About 7.0	0.1C/1.08	100	13
	70.0%	5.0	41.4%	6.4	About 4.5	0.1C/1.08	100	
**V_8C_7–VO_2**	62.8%	9.4	48.9%	5.1	4.17	0.2C/1.7	100	14
	62.8%	5.2	33.2%	9.2	5.11	0.1C/1.5	100	15
**NC/MoS_3**	49.0%	15.0	38.4%	5.5	3.54	0.2C/1.8	100	
**CoSe@HPP**	56.0%	20.0	58.1%	3.7	3.6	0.1C/0.58	30	
	56.0%	15.0	54.3%	4.4	4.0	0.1C/0.7	30	16
	56.0%	7.0	30.3%	8.1	4.1	0.1C/1.35	30	
**LaB_6**	64.0%	14.0	35.9%	3.3	About 2.0	0.5C/2.76	100	17
	64.0%	7.5	39.2%	6.1	4.0	0.2C/2.0	200	
**N-$CoSe_2$**	56.0%	6.0	55.7%	6.37	5.93	0.2C/2.1	70	18
	56.0%	4.4	46.9%	10.2	8.0	0.2C/3.4	70	
**TiO_2–Ni_3S_2**	64.0%	10.2	30.1%	3.92	1.97	0.3C/2.0	500	19
	64.0%	10.2	47.8%	3.92	About 3.1	0.3C/2.0	100	
**Mo_2C/CHS**	53.6%	17.5	49.2%	3.0	2.47	0.3C/1.5	100	20
	53.6%	17.5	46.9%	3.0	2.35	1.0C/5.0	300	
	58.6%	7.0	53.5%	5.0	4.47	0.5C/4.2	200	
**Free-standing $NiCo_2(O\text{-}S)_4$**	**46.5%**	**12.5**	**73.0%**	**3.3**	**3.565**	**0.15C/0.8**	**100**	**This work**
	**40.0%**	**9.52**	**58.5%**	**4.4**	**4.28**	**0.11C/0.8**	**100**	
**Free-standing $NiCo_2(O\text{-}S)_4$ With interlayer**	**51.9 %**	**3.8**	**56.2%**	**8.75**	**8.22**	**0.046C/0.67**	**7**	**This work**
	**51.9 %**	**3.8**	**50.8%**	**8.75**	**7.43**	**0.07C/1.0**	**16**	
	**51.9 %**	**3.8**	**31.0%**	**8.75**	**4.54**	**0.1C/1.33**	**50**	

References

[1] Y. Zhong, L. Yin, P. He, W. Liu, Z. Wu, H. Wang, Journal of the American Chemical Society 2018, 140, 1455.

[2] Y. Song, Z. Wang, Y. Yan, W. Zhao, Z. Bakenov, Microporous and Mesoporous Materials 2021, 316, 110924;

[3] B. Liu, S. Huang, D. Kong, J. Hu, H. Y. Yang, Journal of Materials Chemistry A 2019, 7, 7604;
[4] S. Huang, Y. Wang, J. Hu, Y. Von Lim, D. Kong, L. Guo, Z. Kou, Y. Chen, H. Y. Yang, NPG Asia Materials 2019, 11, 55;
[5] W. Li, S. Li, A. A. Bernussi, Z. Fan, Energy Material Advances 2021, 2021, 2712391;
[6] S. Chen, J. Zhang, Z. Wang, L. Nie, X. Hu, Y. Yu, W. Liu, Nano Letters 2021, 21, 5285;
[7]Y.T. Liu, D.D. Han, L. Wang, G.R. Li, S. Liu, X.P. Gao, Advanced Energy Materials 2019, 9, 1803477.
[8] X. Xia, Materials Research Express 2019, 6, 095309;
[9] Z. Cui, S.-A. He, Q. Liu, R. Zou, Dalton Transactions 2020, 49, 6876.
[10]S. Wang, S. Feng, J. Liang, Q. Su, F. Zhao, H. Song, M. Zheng, Q. Sun, Z. Song, X. Jia, J. Yang, Y. Li, J. Liao, R. Li, X. Sun, Advanced Energy Materials 2021, 11, 2003314;
[11] D. Wang, D. Luo, Y. Zhang, Y. Zhao, G. Zhou, L. Shui, Z. Chen, X. Wang, Nano Energy 2021, 81, 105602;
[12] M. Wang, H. Yang, K. Shen, H. Xu, W. Wang, Z. Yang, L. Zhang, J. Chen, Y. Huang, M. Chen, D. Mitlin, X. Li, Small Methods 2020, 4, 2000353;
[13] H. Ye, J. Sun, S. Zhang, H. Lin, T. Zhang, Q. Yao, J. Y. Lee, ACS Nano 2019, 13, 14208;
[14] J. Cai, J. Jin, Z. Fan, C. Li, Z. Shi, J. Sun, Z. Liu, Advanced Materials 2020, 32, 2005967;
[15] J. Yu, J. Xiao, A. Li, Z. Yang, L. Zeng, Q. Zhang, Y. Zhu, L. Guo, Angewandte Chemie International Edition 2020, 59, 13071;
[16] Z. Ye, Y. Jiang, L. Li, F. Wu, R. Chen, Advanced Materials 2020, 32, 2002168;
[17] J. Cai, Z. Fan, J. Jin, Z. Shi, S. Dou, J. Sun, Z. Liu, Nano Energy 2020, 75, 104970;
[18] M. Wang, L. Fan, X. Sun, B. Guan, B. Jiang, X. Wu, D. Tian, K. Sun, Y. Qiu, X. Yin, Y. Zhang, N. Zhang, ACS Energy Letters 2020, 5, 3041;
[19]R. Wang, C. Luo, T. Wang, G. Zhou, Y. Deng, Y. He, Q. Zhang, F. Kang, W. Lv, Q.H. Yang, Advanced Materials 2020, 32, 2000315.
[20] J. Qian, Y. Xing, Y. Yang, Y. Li, K. Yu, W. Li, T. Zhao, Y. Ye, L. Li, F. Wu, R. Chen, Advanced Materials 2021, 33, 2100810.
